# Oral Copaiba Oil‐Resin Supplementation Reduces Plasma and Hepatic Oxidative Stress in Female and Male Hypothalamic‐Obese Animals

**DOI:** 10.1002/fsn3.72195

**Published:** 2026-09-08

**Authors:** Sirlei P. Souza, Zoé M. N. de Carvalho, Beatriz M. Daudt, Marianela A. F. Urrutia, Caroline M. Aguiar, Ellen C. Z. Gomes, Mustafa M. M. Dahleh, Marina Prigol, Gustavo P. Guerra, Lucinéia F. C. Ribeiro, Sabrina Grassiolli

**Affiliations:** ^1^ Programa de Pós‐graduação em Biociência e Saúde Universidade Estadual do Oeste do Paraná Cascavel PR Brazil; ^2^ Universidade Tecnológica Federal do Paraná Toledo PR Brazil; ^3^ Programa de Pós‐Graduação em Bioquímica Universidade Federal do Pampa Uruguaiana RS Brazil

**Keywords:** liver, natural compounds, obesity, oxidative stress, sex

## Abstract

Oxidative stress is a characteristic of obesity dysfunction‐associated fatty liver disease. Copaiba oil‐resin (Co‐resin) is known for its antioxidant and hepatoprotective properties; however, its effects on the oxidative system of obese rodents are unknown, particularly with regard to potential sex differences. Here, we evaluated the effects of the oral Co‐resin supplementation on plasma and hepatic oxidative parameters in male and female rodents with hypothalamic‐induced obesity. Hypothalamic obesity was induced during the early postnatal days (PND) by monosodium glutamate (MSG; 4 g kg^−1^) administration, while control (CON) animals received equimolar saline solution. From 30 to 90 PND, MSG, and CON groups (both sexes) were divided into Co‐resin‐supplemented (0.5 mL kg^−1^, three times a week/8 weeks) or non‐supplemented animals. Adiposity, plasma, and hepatic metabolic and oxidative markers were assessed. Hepatic tissue was used for histological analysis. Male and female MSG‐obese rats presented higher adiposity and metabolic dysfunction, associated with reductions in plasma and hepatic antioxidative activities of glutathione reductase, glutathione‐S‐transferase, and catalase. Reduced hepatic glutathione peroxidase was also observed in MSG‐obese rats of both sexes. Moreover, plasma, and hepatic thiobarbituric acid reactive substances and reactive species were increased in MSG‐obese (male and female) animals. MSG‐obese rats of both sexes displayed abnormal hepatic histopathology and reduced liver superoxide dismutase and catalase contents. Co‐resin oral supplementation effectively restored plasma and hepatic oxidative systems in MSG‐obese males and females without significantly adiposity change. However Co‐resin supplementation had more positive effects on the metabolic parameters of MSG‐obese females, since it restored liver structural abnormalities, plasma triglyceride levels, and insulin responsiveness.

## Graphical abstract

Oral supplementation with copaiba‐oil resin (Co‐resin) restores hepatic and plasma oxidative systems in hypothalamic‐obese male and female rats, therefore, it showed a more positive impact on females. This study highlights the therapeutic potential of Co‐resin to reduce reactive oxygen species production and restore plasma and hepatic antioxidant systems, thereby, protecting liver from obesity‐induced damage and its metabolic consequences. CAT, catalase; CATWB, catalase Western blot; CP, carbonylated protein; GPx, Co glutathione peroxidase; GR, glutathione; GSH, glutathione reductase; GST, glutathione‐S‐transferase; IR, insulin resistance; MSG, monosodium glutamate; MSG_Co_, obese supplemented with Co‐resin; MSG_NS_, obese non‐SUPPLEMENTED; RS, reactive species; SOD, superoxide dismutase; SODWB, superoxide dismutase Western blot; TBARS, thiobarbituric acid reactive substances. *n* = 10 animals per group. Created in BioRender. Zawoski gomes, E. C. (2026) https://BioRender.com/f8y5ydo.

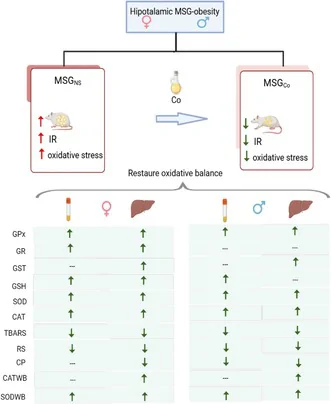

**Abbreviations**
ABTS: 2,2′‐azino‐bis(3‐ethylbenzothiazoline‐6‐sulphonic acid)
ARC: arcuate nuclei
AU: arbitrary units
BW: body weight
CAT: catalase
CATWB: Catalase Western blot
CDNB: 1‐chloro‐2,4‐dinitrobenzene
CEUA: Animal Use Ethics Committee
Co: copaiba oil
CON: control
CONCEA: National Council for the Control of Animal Experiments
CON_Co_
: non‐obese supplemented with Co‐resin
CON_NS_
: non‐obese non‐supplemented
Co‐resin: copaiba‐oil resin
CP: carbonylated protein
DCF: dichlorofluorescein
DCFH‐DA: 2′7′‐dichlorofluorescein diacetate
DPPH: 1,1‐diphenyl‐2‐picrylhydrazyl
F: factors
GA: gallic acid
GAE: gallic acid expressed
GH: growth hormone
GPx: glutathione peroxidase
GR: glutathione
GSH: glutathione reductase
GST: glutathione‐S‐transferase
H_2_O_2_
: hydrogen peroxide
HE: hematoxylin and eosin
I: interaction
IR: insulin resistance
KC: Kupffer cells
LEE: lee index
MASLD: metabolic dysfunction‐associated steatotic liver disease
MSG: monosodium glutamate
MSG_Co_
: obese supplemented with Co‐resin
MSG_NS_
: obese non‐supplemented
NAL: naso‐anal length
NASH: nonalcoholic steatohepatitis
NS: non‐supplemented
PCA: principal component analysis
PND: postnatal day
Q: quercetin
ROS: reactive oxygen species
RS: reactive species
r‐WAT: white adipose tissue retroperitoneal
S1: supernatant fraction
SD: standard deviation
SDS: sodium dodecyl sulfate
SOD: superoxide dismutase
SODWB: Superoxide Dismutase Western blot
TBARS: thiobarbituric acid reactive substances
TF: total flavonoids
TGL: triglycerides
TPC: total phenolic compounds
TyG: triglycerides‐glucose index
WAT: white adipose tissue
WB: Western blot

## 1 Introduction

Obesity is defined by excessive white adipose tissue (WAT) expansion, accompanied by a chronic pro‐inflammatory state that is intimately related to liver injuries. Augmented liver inflammation, with high hepatic fat accumulation and disruption of glycogenolysis and lipogenic pathways, is common in the obesity state (Cusi 2012). These hepatic metabolic disturbances are the primary consequence of hepatic insulin resistance (IR) that directly contributes to hyperglycemia and dyslipidemia, favoring type 2 diabetes mellitus and cardiovascular diseases development (Hashimoto et al. 2013).

Imbalance in oxidation–reduction (redox) reactions, with excessive generation of reactive oxygen species (ROS) is directly involved in metabolic disturbances in obese animals, including liver injuries. Thus, free radicals such as superoxide anions (O_2_^• −^), hydroxyl radical (HO^•^), nitric oxide (^•^NO), and lipid radicals besides non‐radicals such as hydrogen peroxide (H_2_O_2_), peroxynitrite (ONOO¯), and hypochlorous acid (HOCl) generate ROS into cells (Pérez‐Torres et al. 2021). In healthy conditions, several antioxidant enzymes such as superoxide dismutase (SOD), glutathione peroxidase (GPx), glutathione reductase (GR), and catalase (CAT), as well as nonenzymatic compounds such as tocopherol, beta‐carotene, ascorbate, and glutathione (GSH) exert protective actions against excessive ROS production, mitigating their deleterious effects on cellular homeostasis (Valko et al. 2007).

Obesity can overwhelm the capacity of enzymatic and nonenzymatic antioxidants in physiological systems, leading to alterations in redox state and contributing to inflammatory, metabolic, and proliferative liver injuries in obese subjects (Cusi 2012). Moreover, obesity is frequently associated with high consumption of refined carbohydrates and saturated fatty acids, and low intake of dietary fiber, fruits, vegetables, and n‐3 polyunsaturated fatty acids (n‐3 PUFAs), resulting in excessive energy intake. This continuous overnutrition process reduces lipids beta oxidation and stimulates hepatic lipogenesis, resulting in augmented fat liver deposition (Valenzuela et al. 2026). The liver is morphologically constituted by several cell types, including hepatocytes, Kupffer cells (KC), liver sinusoidal endothelial cells, pit cells, and hepatic stellate cells. All of them are negatively impacted by hepatic fat accumulation and inflammation associated with overproduction of ROS (Cusi 2012). Thus, excessive ROS is frequently observed in metabolic dysfunction‐associated steatotic liver disease (MASLD) and is closely related to hepatic IR. These conditions lead to high hepatic fat accumulation, exacerbating steatosis and gradually progressing to complicated nonalcoholic steatohepatitis (NASH) (Hashimoto et al. 2013; Sanyal and Siddiqui 2020). Thus, preserving the potential of the hepatic antioxidant system and controlling hepatic ROS generation are essential to protect liver function and morphology from the deleterious effects associated with the excessive ROS content.

Several diet compounds, such as PUFAs, vitamins, and polyphenols exert anti‐steatotic effects, avoiding or controlling deleterious effects of the ROS in hepatic tissue (Valenzuela et al. 2026). In this regard, many plants contain phytochemicals with bioactive compounds that exert positive effects on health. Among several phytocompounds with broad therapeutic potential, some of them present particular interest such as phenols, ethers, terpenes, and ketones, and many of them present antioxidant properties (Pérez‐Torres et al. 2021; Gonzalez‐Franquesa et al. 2022).

Brazilian rainforest contains an enormous diversity of plants and biomolecules with positive effects on health, such as Copaiba oil (Co), an oil resin extracted from the trunks of trees of *Copaifera* genus (Castro Ghizoni et al. 2017). Co‐resin has been used since the 16th century as an anti‐inflammatory and antiseptic agent. Recently, its application has increased worldwide and in the industry due to its positive health effects (Campos et al. 2021). Several studies have confirmed the benefits of Co‐resin in pathological conditions, reported anti‐inflammatory properties in arthritis, antitumor properties in cancerous cells, and antitetanic, antinociceptive, and antiseptic effects. It is also used to treat bronchitis, syphilis, skin diseases, and ulcers, and many of these benefits are attributed to its antioxidant and anti‐lipoperoxidative properties (da Trindade et al. 2018).

The principal compounds found in Co‐resin are a mixture of different sesquiterpenes, including α‐humulene, β‐caryophyllene, caryophyllene oxide, α‐cadinol, ∆‐cadinene, β‐elemene, β‐bisabolene, α‐cubebene, trans‐α‐bergamotene, α‐selinene, and β‐selinene. Moreover, minor quantities of diterpenes (kaurane, clerodane, or labdane‐type skeletons) can also be found in Co‐resin (Campos et al. 2021; da Trindade et al. 2018). β‐caryophyllene is the most abundant compound in Co‐resin and seems to be important due to its antioxidant actions (Cebollada et al. 2023). Oral Co‐resin administration reportedly also reduces ROS, increases liver GSH content, and CAT activity in arthritic rats (Castro Ghizoni et al. 2017). These studies have also demonstrated that most of these hepatic antioxidant effects were attributable to β‐caryophyllene (Campos et al. 2021; Ames‐Sibin et al. 2018). The hepatoprotective effects of Co‐resin have also been demonstrated in a model of pharmacological agent‐induced hepatic injury (Telles et al. 2022).

Several studies have shown that oral Co‐resin supplementation provides anti‐adiposity benefits (Pérez‐Torres et al. 2021; Telles et al. 2022; Kailer et al. 2024), however, its effects on liver stress during obesity are unknown.

High doses of monosodium glutamate (MSG), when administered during the first postnatal days (PND) in rats, damage the hypothalamic nuclei, particularly the arcuate nucleus (ARC), causing neuroendocrine imbalance and the development of obesity (Elfers et al. 2011; Von Diemen et al. 2006). Hypothalamic MSG‐obese rodents develop excessive adiposity, as well as glucose intolerance, IR, dyslipidemia, inflammation, and liver injury (Balbo et al. 2007; Grassiolli et al. 2006). Augmented hepatic fat accumulation, altered lipogenesis, and gluconeogenesis in hepatocytes have been demonstrated in this model of obesity (Kailer et al. 2024; Sagae et al. 2011). The absence of hyperphagia in MSG‐induced hypothalamic obesity avoids oxidative stress commonly associated with excess caloric intake (Rains and Jain 2011).

Moreover, MSG‐obese rats present hypometabolism, lower skeletal muscle mass, hypercortisolism, and vagal hyperactivity (Zazula et al. 2023). These pathophysiological characteristics, when associated, reflect those of many obese individuals who present sarcopenia (reduced skeletal muscle mass), chronically elevated cortisol levels, and autonomic imbalance (Habboub et al. 2025). Finally, sexual dimorphism is important to determine the degree of liver abnormalities in MASLD‐associated obese state (Teng et al. 2023). In this regard, MSG‐obesity and its associated comorbidities, including oxidative parameters, may be influenced by sex (Quines et al. 2019). So, hypothalamic MSG‐obese rodents reproduce many typical metabolic abnormalities found in human obesity, including MASLD. And, due to its unique metabolic state, this model could provide new information about the beneficial effects of Co‐resin on health. Thus, the current study examined the effects of the oral Co‐resin supplementation on plasma and hepatic oxidative parameters in male and female rodents with hypothalamic‐induced obesity.

## 2 Methods

### 2.1 Type of Study and Ethical Aspects

The present study is experimental, quantitative, and analytical in nature and was developed at the Study Laboratories of the Western Paraná State University (UNIOESTE). This research project was in line with the Brazilian federal law, established by the National Council for the Control of Animal Experiments (CONCEA) and was previously approved by the Animal Use Ethics Committee (CEUA) of UNIOESTE (Protocol 13/20). It also follows the animal research principles defined by the ARRIVE Guidelines. Female (♀) and male (♂) and Wistar rats were divided into non‐obese and obese groups and were distributed into Co‐resin supplemented or non‐supplemented (NS) subgroups, according to the experimental design and protocols described below.

### 2.2 Animals

Ten pregnant rats were acquired from the central animal facility and transferred to a local facility, where they were maintained under controlled photoperiod (12 h light–dark cycle) and temperature (23°C ± 3°C) conditions. They received free access to rodent chow and water until the birth of offspring. On the first PND, the number of offspring per litter was adjusted to 6–8 pups per dam with similar rates between the sexes (3–4 ♀ and 3–4 ♂). From the 2nd to 6th PND, half pups (♀ and ♂) received high subcutaneous doses of monosodium L‐glutamate (4 g kg^−1^) to induce hypothalamic obesity, according to the previous description of Olney (Olney 1969), with adaptations (Balbo et al. 2007; Grassiolli et al. 2006). During the same period, the other half (♀ and ♂) received equimolar sodium chloride solution (NaCl 1.25 g kg^−1^) administrations and were denominated as non‐obese, or Control (CON), animals. At PND 21, MSG and CON animals of both sexes were weaned, randomly transferred to cages (3–4 rats) according to sex (♀ and ♂) and submitted to the supplementation protocol described below.

### 2.3 Co‐Resin Supplementation

From 30 PDN, subgroups of CON and MSG animals (♀ and ♂) started to receive Co‐resin oral supplementation (0.5 mL kg^−1^ Co‐resin) (AmazonOil) three times‐a‐week for 8 weeks. This dose was defined by previous studies (Kailer et al. 2024; Bahr et al. 2018; Guareschi et al. 2025). The remainder of CON and MSG animals of both sexes formed the NS groups, which received by the same via, period and frequency just a saline (0.9%) solution. All animals were evaluated at PND 92 and 48 h after the last Co‐resin dose.

### 2.4 Experimental Design

**FIGURE 1:**
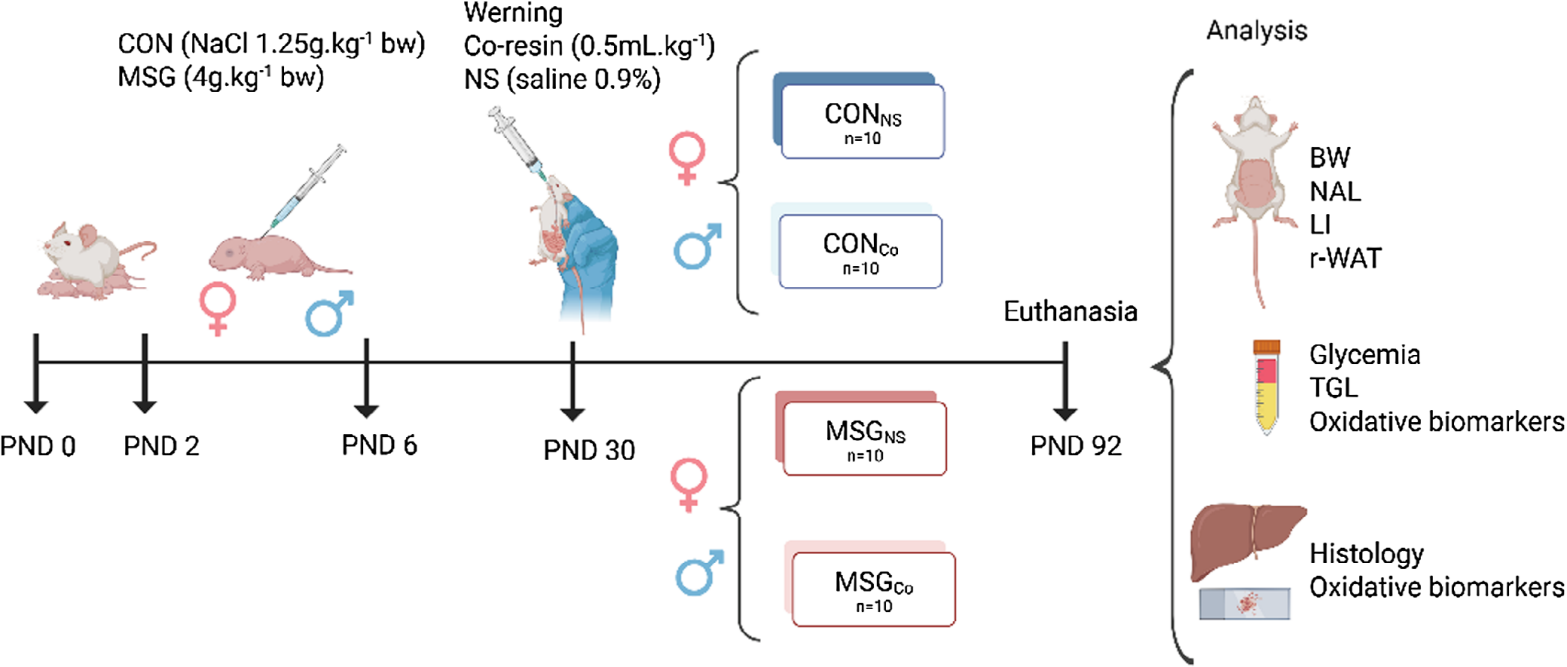
Experimental design. BW, body weight; Co MSG_NS_‐♀, obese non‐supplemented female; Co, copaiba oil; CON, non‐obese; CON_Co_‐♀, non‐obese supplemented with Co‐resin female; CON_Co_‐♂, non‐obese supplemented with Co‐resin male; CON_NS_‐♀, non‐obese non‐supplemented female; CON_NS_‐♂, non‐obese non‐supplemented male; LI, Lee index; MSG, monosodium glutamate; MSG_Co_‐♀, obese supplemented with Co‐resin female; MSG_Co_‐♂, obese supplemented with Co‐resin male; MSG_NS_‐♂, obese non‐supplemented male; NAL, naso‐anal length; NS, unsupplemented; PND, postnatal day; r‐WAT, white adipose tissue retroperitoneal; TGL, triglycerides (*n* = 10 animals per group). Created in BioRender. Zawoski Gomes, E. C. (2026), https://BioRender.com/undefined.

Considering obesity (MSG and CON), sexes (♀ and ♂) and supplementation (Co and NS), eight experimental groups were generated in the present study (*n* = 8–10 rats per group): CON_NS_‐♀: control non‐supplemented females; CON_Co_‐♀: control Co‐supplemented females; MSG_NS_‐♀: obese non‐supplemented females; MSG_Co_‐♀: obese Co‐supplemented females; CON_NS_‐♂: control non‐supplemented males; CON_Co_‐♂: control Co‐supplemented males; MSG_NS_‐♂: obese non‐supplemented males; MSG_Co_‐♂: obese Co‐supplemented males, as shown in Figure 1.

### 2.5 Co‐Resin Composition and Determination of Antioxidant Activity (In Vitro) and Antioxidant Compounds

The Co‐resin was donated by the Amazon Oil Company, and its principal compounds may be accessed at https://amazonoil.com.br/produtos‐da‐floresta/oleo‐resina‐de‐copaibacopaifera‐officinalis/. We also carried out an analysis of total phenolic compounds (TPC) using the Folin‐Ciocalteu method with modifications (Boroski et al. 2015). The amount of TPC was calculated as gallic acid (GA) equivalent to the calibration curve of standard GA solutions and expressed as mg GAE g^−1^. The total flavonoids (TF) content of Co‐resin was determined by the aluminum chloride method (Dolce and Della 2023). The amount of TF was determined as quercetin (*Q*) equivalent to the calibration curve of standard *Q* solutions and expressed as mg QE g^−1^. All measurements of TPC and TF were performed in triplicate samples. Were also evaluated in Co‐resin the *free radical scavenging ability* using DPPH (1,1‐diphenyl‐2‐picrylhydrazyl) and ABTS [2,2′‐azino‐bis (3‐ethylbenzothiazoline‐6‐sulphonic acid)] assays, as described by Boroski et al. (2015) with modifications, using Trolox as standard. The results of the antioxidant activity were expressed in μmol of Trolox g^−1^.

### 2.6 Euthanasia, Biometric, and Metabolic Parameters

Body weight (BW; g) was registered at PND 92, and after 12 h of fasting. The rats were briefly desensitized in a carbon dioxide (CO_2_) chamber to avoid the well‐recognized effects of anesthetics such as, xylazine and ketamine on metabolic and hormonal parameters (Saha et al. 2005; De Oliveira et al. 2013). Subsequently, the naso‐anal length (NAL; cm) was immediately recorded to calculate the Lee Index (LI) (Bernardis and Patterson 1986; Jagot et al. 1980) and the rat was decapitated. After decapitation, the total blood volume was collected in heparinized tubes. Blood was then centrifuged (3000 rpm min^−1^; 15 min) and plasma was stored at −20°C for subsequent measurements of glucose (mg dL^−1^) and triglycerides (TGL; mg dL^−1^) using colorimetric enzymatic kits (Lab Test, BR; GPO‐Trinder, BR, respectively) and following the manufacturers' instructions. IR was determined from the fasting values of glucose and triglycerides using triglycerides‐glucose index (TyG) (Simental‐Mendía et al. 2008). Plasma antioxidant activity was also evaluated as described below. The abdominal cavity was then laparotomized, and WAT from retroperitoneal (r‐WAT) depot was excised and weighed, whose result was expressed in g 100 g^−1^ BW. Liver was removed and weighed (g 100 g^−1^ of BW), and a fragment of it was used for enzymatic and molecular analysis. Other fragments were used for histological procedures, as described in the subsequent sections.

### 2.7 Analysis of Liver and Plasma Antioxidant Activities

Approximately 1 g of liver was homogenized in a Tris‐HCl buffer (50 mM, pH 7.4). The resulting homogenate was centrifuged at 10,000× *g* for 10 min at 4°C, and the supernatant fraction (S1) was collected for the analysis of oxidative stress markers and antioxidant enzyme activity. Similarly, S1 from non‐hemolyzed plasma was used for biochemical assays, except for GSH and Western blot analysis (WB).

### 2.8 Glutathione Peroxidase Activity

The measurement of GPx activity was carried out spectrophotometrically, following the procedure by Wendel (1981). This method involves the oxidation of GSH to oxidized glutathione and dismutation of H_2_O_2_ in water. GPx activity was indirectly determined by monitoring NADPH decay upon addition to the reaction, which is catalyzed by GR and results to form GSH and NADP^+^. In this assay, 800 μL reductase system (NADPH 0.15 mM, GSH 1 mM, 10 μL GR (500 U), and 250 μL azide 100 mM dissolved in 20 mL of TFK buffer 100 mM, pH 7.0) were mixed with 50 μL S1 (liver or plasma) and 100 μL H_2_O_2_ (4 mM). The absorbance of samples was read at 30‐s intervals for 2 min using a spectrophotometer at 340 nm. The results were expressed in nmol NADPH min^−1^ mg^−1^ protein.

### 2.9 Glutathione Reductase Activity

GR activity was measured following the method described by Carlberg and Mannervik (1985), which is based on the NADPH consumption, according to GR activity. In this assay, 750 μL of reductase system (0.15 M TFK/EDTA 1.5 mM, pH 7.0, and NADPH 0.15 mM) were combined with 100 μL S1 (liver or plasma). Spectrophotometry was carried out at 340‐nm wavelength, at 30‐s intervals during 2 min. Then, 50 μL of glutathione disulfide were added, and the spectrophotometer was used to analyze the 340‐nm wavelength at 30‐s intervals for two more minutes. A duplicate of each sample was carried out, and the mean value was calculated. The difference between both absorbances was determined, and the results were expressed in nmol NADPH min^−1^ mg^−1^ protein.

### 2.10 Glutathione‐S‐Transferase Activity

Glutathione‐S‐transferase (GST) activity was assayed by measuring the conjugation of glutathione with 1‐chloro‐2,4‐dinitrobenzene (CDNB), as described by Habig et al. (1976). GST is an important phase II enzyme involved in detoxification of xenobiotic substances. Thus, in order to perform the assay, 50 μL S1 (liver or plasma) were mixed with 0.1 M potassium phosphate buffer (pH 7.5), 50 mM CDNB as the substrate, and 100 mM GSH to initiate the reaction. The absorbance of samples was read at 30‐s intervals for 2 minusing a 340‐nm spectrophotometer. The results were expressed in μmol min^−1^ mg^−1^ protein.

### 2.11 Glutathione Levels

The determination of total glutathione (GSH) levels was carried out following the method described by Hissin and Hilf (1976). Liver was homogenized in 0.1 M HCl_4_, and homogenates were centrifuged at 2500× g for 10 min at 4°C to obtain supernatants. A 100‐μL portion of supernatant was mixed with 800 μL of TFK buffer (0.1 M, pH 8.0) and 100 μL of ortho‐phthalaldehyde as a fluorophore. Plasma GSH analysis consisted of adding samples to 0.1 M HCl_4_, 800 μL of TFK buffer (0.1 M, pH 8.0) and 100 μL of ortho‐phthalaldehyde. The samples were incubated in the dark for 15 min. The samples analysis was carried out using a fluorescence spectrophotometer with an excitation wavelength of 350 nm and an emission wavelength of 420 nm. The results were expressed in nmol GSH mg^−1^ tissue, based on the mean value.

### 2.12 Superoxide Dismutase Activity

SOD activity was measured according to the method outlined by Misra and Fridovich (1972). To start the reaction, aliquots of S1 (liver or plasma; ranging from 6 to 18 μL) were combined with 30 μL of a 6 mM epinephrine substrate in a solution containing aliquots (ranging from 260 to 242 μL) of a 57.7 mM sodium carbonate buffer at 30°C. Absorbance readings were taken at 10‐s intervals for 2 min using a spectrophotometer set to a 480‐nm wavelength. One unit of enzyme activity was defined as the amount of enzyme necessary to inhibit 50% of epinephrine oxidation at 30°C. Results were presented as units per mg of protein.

### 2.13 Catalase Activity

CAT activity was assessed following the method proposed by Aebi (1984), which is based on the breakdown of H_2_O_2_ in the presence of S1 (liver or plasma), with absorbance monitored at 240 nm. To start the reaction, 20 μL of S1 were mixed with 105 μL of 0.3 mM H_2_O_2_ (substrate) in a solution containing 100 μL of 50 mM phosphate buffer at pH 7.0. The results were reported in enzyme units, where one unit is defined as the amount of enzyme required to decompose 1 μmole of H_2_O_2_ min^−1^ at pH 7.0 and 25°C.

### 2.14 Thiobarbituric Acid‐Reactive Substances Levels

Lipid peroxidation was assessed by quantifying thiobarbituric acid reactive substances (TBARS) levels following the method outlined by Ohkawa et al. (1979). In brief, 200 μL of S1 (liver or plasma) were combined with 500 μL of 0.8% TBA, 200 μL of 8.1% sodium dodecyl sulfate (SDS), and 500 μL of acetic acid. The mixture was then incubated at 95°C for 2 h. After incubation, absorbance was measured at 532 nm, and the results were expressed as nmol MDA mg^−1^ protein.

### 2.15 Reactive Species Levels

The measurement of reactive species (RS) production was carried out following the protocol outlined by Loetchutinat et al. (2005). In summary, 10 μL of S1 (liver or plasma) were combined with 3000 μL of Tris‐HCl (10 mM, pH 7.4). The reaction was initiated by introducing 1 mM of 2′7′‐dichlorofluorescein diacetate (DCFH‐DA), a nonfluorescent compound that easily penetrates cell membranes and rapidly undergoes oxidation to form dichlorofluorescein (DCF), a fluorescent derivative, in the presence of RS. After 1 h, the fluorescence emission of DCFH‐DA was measured at an excitation wavelength of 488 nm and an emission wavelength of 520 nm using a fluorescence spectrometer. The results were obtained and expressed as a percentage of the control DCF formation in arbitrary units (AU).

### 2.16 Protein Carbonyl Content

In protein carbonyl content analysis, we employed the methodology described by Reznick (1994) with minor modifications. The procedure involved dinitrophenylhydrazone detection, a result from the reaction between carbonylated proteins (CP) and dinitrophenylhydrazine. In this analysis, we used 400 μL S1 (liver or plasma) solution along with 200 μL of 10 mM 2,4‐dinitrophenylhydrazine prepared from 2 M HCl. Samples were incubated in darkness for 60 min, with intermittent shaking every 15 min. So, according to the incubation period, we added 500 μL of denaturation buffer (composed of 150 mM sodium phosphate at pH 6.8 and 0.1 M SDS), along with 1500 μL of ethanol and 1500 μL of hexane to the samples. Homogenization was carried out for 40 s, and subsequently, the samples were centrifuged at 2400× *g* at 4°C. The supernatant was discarded, and the resulting pellet was submitted to two washes with 1000 μL of ethanol/ethyl acetate (1:1). Finally, the samples were suspended again in 1000 μL denaturation buffer and analyzed using a spectrophotometer at a 370‐nm wavelength. The results were reported in nmol carbonyl mg^−1^ protein.

### 2.17 Liver and Plasma SOD and CAT Western Blot Analysis

Western blotting analysis was carried out following the protocol described by Guerra et al. (2012). Thus, a fragment of liver was promptly excised after decapitation and homogenized in 300 μL of ice‐cold buffer A (10 mM KCl, 2 mM MgCl_2_, 1 mM EDTA, 1 mM NaF, 10 μg mL^−1^ aprotinin, 10 mM β‐glycerolphosphate, 1 mM PMSF, 1 mM DTT, and 2 mM sodium orthovanadate in 10 mM HEPES pH 7.9). Plasma samples were directly added and mixed into ice‐cold buffer A. The homogenate was then centrifuged at 16,000× *g* for 45 min at 4°C. Protein concentration was determined using Bradford reagent, and equal amounts of protein (80 μg) were combined with 0.2 volumes of concentrated loading buffer. The loading buffer contained 200 mM Tris, 10% glycerol, 2% SDS, 2.75 mM β‐mercaptoethanol, and 0.04% bromophenol blue, and samples were incubated at 100°C for 5 min.

The obtained proteins were separated using 12% SDS‐PAGE gels for approximately 3 h. Subsequently, the gel was transferred onto Amersham Protran Premium WB nitrocellulose membranes using the Transfer‐Blot Turbo Transfer System at a constant current of 1.0 mA for 30 min. After blocking for 2 h with 1% bovine serum albumin in 0.05% TBS‐Tween 20. The membranes were incubated overnight with specific primary polyclonal antibodies, anti‐rabbit MnSOD and CAT (1:1000; Sigma‐Aldrich; St. Louis, MO, USA) and loading control anti‐mouse β‐actin (1:1000; Sigma‐Aldrich, St. Louis, USA). The membranes were washed three times with 0.05% TBS‐T and subsequently incubated with a secondary antibody conjugated with horseradish peroxidase (1:5000, anti‐rabbit IgG; Santa Cruz Biotechnology Inc., Santa Cruz, CA, USA) for 2 h at room temperature. Subsequently, the membranes were developed using 3,3′,5,5′‐Tetramethylbenzidine (TMB; Sigma‐Aldrich; St. Louis, MO, USA) and quantified using ImageJ software (NIH, Bethesda, MD, USA). The results were normalized by arbitrarily establishing the ratio between the protein band intensity and the β‐actin control. Uncropped WB membranes were available as supplementary data (Figure S1: SOD and CAT; Figure S2: β‐actin).

### 2.18 Liver Histology

After euthanasia, a fragment of liver (*n* = 6 rats per group) was excised and immediately transferred to Metacarn fixation solution (70% methanol + 20% chloroform + 10% glacial acetic acid) for 24 h. Afterwards, the stored tissue samples underwent dehydration in a graded alcohol series, followed by diaphanization in n‐butyl alcohol, and inclusion in histological paraffin. The 5 μm‐thick sections were stained with hematoxylin and eosin (HE) and analyzed by light microscopy to observe the standard morphological characteristics of tissues. The slides were photomicrographed (40×) for morphometric analyses of the liver tissue. So, 10 photos were obtained per section for quantitative analysis using the Image Pro Plus 6.0 program.

A qualitative assessment was carried out to check for hepatic steatosis, based on a score for nonalcoholic fatty liver disease, in which steatosis is classified using hepatocytes percentage that contained lipid droplets, where < 5% (Grade 0); 5% to 33% (Grade 1); > 33% to 66% (Grade 2) and > 66% (Grade 3) (Kleiner et al. 2005). Representative sections from each group were photomicrographed and presented in the form of histomorphological plates to demonstrate tissue morphology.

### 2.19 Statistical Analysis

The results were expressed as mean ± standard deviation (SD) when data normality was accepted. Normality was assessed using the Shapiro‐Wilk test, then, the two‐way ANOVA was applied to obtain *p* values for factors (F) Co‐resin supplementation (Co); MSG obesity (M) and interaction (I). When *F* was significant, Tukey‐HSD post‐hoc test was used. The adopted significance was 5% and analyses were made using GraphPad Prism software.

### 2.20 Principal Component Analysis (PCA)

Considering the complexity of data in the present study, we also applied PCA to cluster variables of the oxidative system and simplify interpretation of complex datasets. Thus, PCA was implemented in R environment using the “FactoMineR” package. For this, a data matrix was created containing, as variables, “Glutathione peroxidase” (GPx), “Glutathione reductase” (GR), “Glutathione‐S‐transferase” (GST), “Reduced glutathione” (GSH), “Superoxide dismutase” (SOD), “Catalase” (CAT), “Thiobarbituric acid” (TBARS), “Reactive species” (RS), “carbonylated protein” (CP), “SOD Western blot” (SODWB) and “CAT Western blot” (CATWB). The auto‐values were then calculated to obtain dimensions (Dim.) and their contribution to the total variability of data. A graph of ordering autovectors of PCA was created with the “ggplot2” package, using ellipses to visually highlight the groups. Factorial loads were analyzed for normality and homoscedasticity of residues, using Shapiro‐Wilk and Bartlett tests. Two‐factor variance analysis (Two‐Way ANOVA) was carried out to test the significance of factorial loads with application of Tukey‐HSD post‐hoc test (*p* < 0.05). All analyses were carried out using R Core Team (2024) software, version 4.4.0.

## 3 Results

### 3.1 Antioxidant Properties and Activity of Co‐Resin (In Vitro)

The analysis of Co‐resin effects showed high antioxidant potential, as described in Table 1, with 0.605 mg GAE g^−1^ (±1.77) of CFT and 0.442 mg QE g^−1^ (±0.13) of FT. The antioxidant activity values were 6129.97 μmol Trolox g^−1^ (±37.87) and 45,096.67 μmol Trolox g^−1^ (±21.29), for DPPH and ABTS respectively. These answers showed that this oil presents antioxidant compounds, as well as antioxidant activity.

**TABLE 1:** Phenolic compounds, flavonoid content and antioxidant activity (DPPH and ABTS) of Co.

TPC (mg GAE g^−1^)	TF (mg QE g^−1^)	DPPH (μmol Trolox g^−1^)	ABTS (μmol Trolox g^−1^)
0.605 ± 1.77	0.442 ± 0.13	6129.97 ± 37.87	45,096.67 ± 21.29

*Note:* Data are presented as mean ± SD.

Abbreviations: ABTS, 2,2′‐azino‐bis (3‐ethylbenzothiazoline‐6‐sulfonic acid); DPPH, 1,1‐diphenyl‐2‐picrylhydrazyl; TF, total flavonoids; TPC, total phenolic compounds.

### 3.2 Effects of Co‐Resin Supplementation on Biometric Parameters, Adiposity and Plasma Biochemistry Variables of Hypothalamic Obese (MSG) and Non‐obese (CON) Females (Table 2)

Treatment of neonates with MSG significantly influenced final BW (*p* < 0.0001), NAL (*p* < 0.0001), LI (*p* < 0.0001), r‐WAT (*p* = 0.011), fasting glycemia (*p* = 0.039), TGL (*p* = 0.0002) and the TyG index (*p* = 0.0015) in females (Table 2). Thus, lower BW (24.31% and 27.25%), NAL (16.67% and 17.17%) and higher LI (9.68% and 6.45%) were observed in MSG_NS_ and MSG_Co_ female groups, respectively, in relation to CON_NS_ females. Moreover, MSG_NS_ females showed higher r‐WAT content (95%), when compared with CON_NS_ female group. The females in MSG_NS_ group presented higher fasting values of glucose (13.91%) and TGL (44.06%), in relation to the other groups. As a consequence, elevated values of TyG index were observed in MSG_NS_ female group, in comparison to CON_NS_ and CON_Co_ animals. Additionally, Co (*p* = 0.0060) and I (*p* = 0.0002) effects were observed for TGL values, with MSG_Co_ females and presented similar fasting values for TGL to those of non‐obese groups (CON_NS_ and CON_Co_).

**TABLE 2:** Effects of chronic supplementation with Co on growth, adiposity and metabolic parameters of hypothalamic‐obese (MSG) and non‐obese (CON) females.

	Females (♀)				*F* (*p*)		
	CON_NS_	MSG_NS_	CON_Co_	MSG_Co_	I	Co	M
Final BW (g)	225.60 ± 12.22^b,d^	170.75 ± 18.98^a,c^	214.90 ± 15.34^b,d^	164.12 ± 15.34^a,c^	0.10 (0.7501)	1.84 (0.1837)	69.83 (< 0.0001)
NAL (cm)	19.8 ± 1.30^b,d^	16.5 ± 1.00^a,c^	18.7 ± 0.63^b,d^	16.4 ± 0.74^a,c^	1.89 (0.1841)	2.95 (0.0988)	62.41 (< 0.0001)
LI	0.31 ± 0.02^b,d^	0.34 ± 0.01^a,c^	0.32 ± 0.01^b,d^	0.33 ± 0,01^a,c^	1.43 (0.2418)	0.81 (0.3763)	12.68 (0.0017)
r‐WAT (g 100 g^−1^ bw)	0.20 ± 0.07	0.39 ± 0.24^c^	0.16 ± 0.12^b^	0.27 ± 0.10	0.46 (0.5055)	2.21 (0.1501)	7.62 (0.0111)
Glucose (mg dL^−1^)	94 ± 7.24^b^	109.2 ± 8.18^a,c,d^	96.2 ± 13.15^b^	98.9 ± 6.85^b^	2.36 (0.1393)	0.98 (0.3298)	4.77 (0.0395)
TGL (mg dL^−1^)	47.09 ± 6.68^b^	84.18 ± 23.69^a,c,d^	45.96 ± 9.57^b^	54.30 ± 11.28^b^	7.87 (0.0100)	9.17 (0.0060)	19.67 (0.0002)
TyG	4.19 ± 0.06^b^	4.44 ± 0.25^a,c^	4.18 ± 0.08^b^	4.29 ± 0.10	2.13 (0.1539)	2.63 (0.1160)	13.02 (0.0015)

*Note:* Data are presented as mean ± SD (*n* 4–10 rats per group) submitted to a two‐way ANOVA analysis of variance for analysis of *p* values of factors (*F*): Co (copaiba oil), MSG (obesity) and I (interaction). When *F* was significant, the means among groups were compared with Tukey's posttest (*p* < 0.05), with statistical differences represented by letters over numbers (a, CON_NS_; b, MSG_NS_; c, CON_CO_; d, MSG_Co_).

### 3.3 Effects of Co‐Resin Supplementation on Biometric Parameters, Adiposity, and Plasma Biochemistry Variables in Hypothalamic Obese (MSG) and Non‐obese (CON) Males; Table 3

Neonatal treatment with MSG significantly influenced BW (*p* < 0.0001), NAL (*p* < 0.0001), LI (*p* < 0.0001), r‐WAT (*p* = 0.0002), fasting glycemia (*p* < 0.0001), TGL (*p* = 0.0002) and the TyG index (*p* < 0.0001) in males (Table 3). Here, we also observed I (Co × M) effects (*p* = 0.0396) on NAL group. Thus, the males in the MSG_NS_ and MSG_Co_ groups showed lower BW (28.44% and 28.67%), NAL (20.61% and 19.30%), in addition to higher LI (9.68% and 9.68%) and r‐WAT content (67.98% and 92.61%), in relation to CON_NS_ group. Furthermore, MSG_NS_ and MSG_Co_ males also presented higher values for glycemia (21.87% and 14.46%), TGL (73.47% and 109.67%) and TyG (8.75% and 10.16%), when compared with CON_NS_ group.

**TABLE 3:** Effects of chronic Co supplementation on growth, adiposity, and metabolic parameters of hypothalamic‐obese (MSG) and non‐obese (CON) males.

	Males (♂)				*F* (*p*)		
	CON_NS_	MSG_NS_	CON_Co_	MSG_Co_	I	Co	M
Final BW (g)	339.60 ± 15.98^b,d^	243 ± 40.12^a,c^	315.62 ± 17.39^b,d^	242.25 ± 19.35^a,c^	1.51 (0.2301)	1.72 (0.2024)	81.84 (< 0.0001)
NAL (cm)	22.8 ± 0.27^b,d^	18.1 ± 1.49^a,c^	21.5 ± 0.59^b,d^	18.4 ± 0.98^a,c^	4.81 (0.0396)	1.79 (0.1934)	110.80 (< 0.0001)
LI	0.31 ± 0,01^b,d^	0.34 ± 0.01^a,c^	0.32 ± 0.01^b,d^	0.34 ± 0.01^a,c^	3.25 (0.0846)	0.35 (0.5610)	41.87 (< 0.0001)
r‐WAT (g 100 g^−1^ BW)	0.203 ± 0.03^d^	0.341 ± 0.08^c^	0.156 ± 0.07^b,d^	0.391 ± 0.14^a,c^	1.42 (0.2456)	0.00 (0.9825)	21.19 (0.0002)
Glucose (mg dL^−1^)	87.8 ± 5.54^b^	107 ± 0.83^a,c^	86.57 ± 0.27^b,d^	100.5 ± 8.37^c^	0.63 (0.4336)	1.41 (0.2445)	25.98 (< 0.0001)
TGL (mg dL^−1^)	55.75 ± 16.14^d^	96.71 ± 12.27	63.05 ± 9.29^d^	116.89 ± 38.95^a,c^	0.39 (0.5384)	1.79 (0.1964)	21.19 (0.0002)
TyG	4.23 ± 0.12^b,d^	4.6 ± 0.10^a,c^	4.29 ± 0.10^b,d^	4.66 ± 0.18^a,c^	0.04 (0.8454)	0.88 (0.3563)	43.02 (< 0.0001)

*Note:* Data are presented as mean ± SD (*n* 4–10 rats per group) submitted to a two‐way ANOVA analysis of variance for analysis of *p* values of factors (*F*): Co (copaiba oil), MSG (obesity) and I (interaction). When *F* was significant, the means among groups were compared with Tukey's posttest (*p* < 0.05), with statistical differences represented by letters over numbers (a, CON_NS_; b, MSG_NS_; c, CON_Co_; d, MSG_Co_).

### 3.4 Effects of Co‐Resin Supplementation on Antioxidant Enzyme Activities and Oxidative Stress Biomarkers in Plasma (Table 4) and Liver (Table 5) of Hypothalamic‐Obese (MSG) and Non‐obese (CON) Females

MSG‐induced obesity had a significant effect on plasma antioxidant enzyme activities of GR (*p* < 0.0001), GST (*p* < 0.0001), and CAT (*p* < 0.0001) in female rats. Thus, MSG_NS_ females presented reductions in plasma activities of GR (78.49%), GST (3.57%), and CAT (81.74%), in comparison to plasma enzymes in CON_NS_ females. Moreover, plasma concentrations of TBARS (*p* = 0.0089) and RS (*p* = 0.048) were also influenced by the MSG effect, with the MSG_NS_ females presenting higher plasmatic TBARS (75.19%) and RS (42.92%) levels when compared with those CON_NS_ females (Table 4).

**TABLE 4:** Effects of chronic supplementation with Co on indicators of oxidative stress in plasma of MSG‐obese and non‐obese (CON) females.

	Females (♀)				*F* (*p*)		
	CON_NS_	MSG_NS_	CON_Co_	MSG_Co_	I	Co	M
GPx (nmol NADPH min^−1^ mg^−1^ protein)	4.64 ± 2.53^b^	1.58 ± 0.31^a,c^	3.66 ± 0.65^b^	5.04 ± 2.02^b^	7.46 (0.0142)	2.31 (0.1467)	1.06 (0.3174)
GR (nmol NADPH min^−1^ mgf^−1^ protein)	19.57 ± 1.90^b,c^	4.21 ± 3.08^a,c,d^	18.06 ± 0.60^b^	11.83 ± 3.90^a,b^	9.88 (0.0059)	4.43 (0.0504)	55.27 (< 0.0001)
GST (μmol min^−1^ mg^−1^ protein)	3.92 ± 1.41^b,d^	3.78 ± 1.00^a,c^	1.28 ± 0.32^b^	2.21 ± 0.22^a^	1.81 (0.1967)	1.01 (0.3291)	28.70 (< 0.0001)
GSH (nmol GSH mg^−1^ of tissue)	10.48 ± 0.64^b^	4.26 ± 1.48^a,c,d^	10.95 ± 2.06^b^	12.77 ± 5.76^b^	7.04 (0.0167)	8.77 (0.0087)	2.12 (0.1640)
SOD (units mg^−1^ protein)	10.77 ± 0.96^b^	8.30 ± 0.68^a,d^	10.70 ± 0.17	11.30 ± 1.89^b^	7,71 (0.0129)	7.04 (0.0167)	2.86 (0.1091)
CAT (units mg^−1^ protein)	17.69 ± 4.16^b,d^	3.23 ± 1.89^a,c,d^	19.34 ± 3.18^b,d^	10.33 ± 3.33^a,b,c^	3.37 (0.0841)	8.68 (0.009)	62.40 (< 0.0001)
TBARS (nmol MDA mg^−1^ protein)	3.87 ± 1.58^b^	6.78 ± 1.10^a,c,d^	4.13 ± 0.74^b^	4.33 ± 0.75^b^	6.66 (0.0195)	4.38 (0.0516)	8.73 (0.0089)
RS (% DCF of Arbitrary Unit)	100 ± 27.89^b^	142.92 ± 21.39^a,c,d^	88.27 ± 18.10^b^	87.10 ± 14.00^b^	5.08 (0.0377)	11.91 (0.0030)	4.55 (0.0478)
CP (nmol CP mg^−1^protein)	3.51 ± 1.19	3.51 ± 1.19	2.06 ± 0.61	2.06 ± 0.61	0.00 (> 0.9999)	7.55 (0.0157)	0.00 (> 0.9999)

*Note:* Data are presented as mean ± SD (*n* 3–6 rats per group) submitted to a two‐way ANOVA analysis of variance for analysis of *p* values of factors (*F*): Co (copaiba oil), MSG (obesity) and I (interaction). When *F* was significant, the means among groups were compared with Tukey's posttest (*p* < 0.05), with statistical differences represented by letters over numbers (a, CON_NS_; b, MSG_NS_; c, CON_Co_; d, MSG_Co_).

Co‐resin supplementation influenced plasma enzyme activities for GR (*p* = 0.0504), GSH (*p* = 0.0087), SOD (*p* = 0.0167), and CAT (*p* = 0.009). Moreover, a significant I (Co × M) effect was noted for GPx (*p* = 0.0142), GR (*p* = 0.0059), GSH (*p* = 0.0167), and SOD (*p* = 0.0129). Therefore, the plasma of females in the MSG_Co_ supplemented group demonstrated augmented activities of GPx (68.65%), GR (64.41%), GSH (66.64%), SOD (26.55%), and CAT (68.73%), compared with the same enzymes in the MSG_NS_ animals.

Similarly, RS plasmatic levels were significantly influenced by M (*p* = 0.0478), Co (*p* = 0.0030), and I (Co × M) effects (*p* = 0.0377). We also noted I effects (Co × M) on plasmatic TBARS (*p* = 0.0195) levels. Thus, smaller values of TBARS (36.13%) and RS (39.05%) were observed in plasma of MSG_Co_ supplemented females in relation to the MSG_NS_ female group (Table 4).

Effects of MSG influenced hepatic activities of GPx (*p* = 0.017), GR (*p* < 0.0001), GST (*p* = 0.0012), GSH (*p* = 0.0004), and CAT (*p* = 0.0002) in females with MSG_NS_ group demonstrating reduced activities of GPx (80.14%), GR (74.72%), GST (56.59%), GSH (59.03%), and CAT (74.03%) in relation to CON_NS_ females. Similarly, effects of MSG were noted on TBARS (*p* = 0.0004), RS (*p* = 0.048), and CP (*p* < 0.0001). Thus, in the liver of MSG_NS_ were found higher levels of TBARS (77.21%), RS (46.83%), and CP (242.42%), compared with the livers of the CON_NS_ group.

Co‐resin supplementation exerted a significant effect on hepatic antioxidant‐enzymatic influencing GR activity (*p* = 0.006) and CAT (*p* = 0.001). Also noted were the I (Co x M) effects to GPx (*p* = 0.036), GST (*p* = 0.002), and SOD (*p* = 0.0013). Therefore, the livers of MSG_Co_ supplemented females demonstrated augmented activities of GPx (80.14%), GR (28.92%), CAT (11.05%), GST (56.59%), SOD (37.41%), and CAT (11.05%) in relation to the females' livers in the MSG_NS_ group.

In addition, Co‐resin supplementation reduced hepatic TBARS (*p* = 0.001) and CP levels (*p* = 0.0003) in females. Moreover, I (Co × M) effects were also found on TBARS (*p* = 0.020), RS (*p* = 0.054), and CP content (*p* = 0.0003). Thus, hepatic tissue of MSG_Co_ supplemented females presented reductions in the presence of TBARS (40.95%), RS (31.46%), and CP content (50%), in comparison with females' livers in the MSG_NS_ group (Table 5).

**TABLE 5:** Effect of chronic supplementation with Co on oxidative stress indicators for liver in MSG‐obese and non‐obese females.

	Females (♀)				*F* (*p*)		
	CON_NS_	MSG_NS_	CON_Co_	MSG_Co_	I	Co	M
GPx (nmol NADPH min^−1^ mg^−1^ protein)	32.73 ± 17.40^b^	6.50 ± 1.31^a,c,d^	29.33 ± 18,66^b^	27.35 ± 5.77^b^	5.10 (0.0358)	2.64 (0.1206)	6.90 (0.0166)
GR (nmol NADPH min^−1^ mgf^−1^ protein)	31.53 ± 13.7^b^	7.97 ± 2.62^a,b,c^	36.06 ± 7.67^b,c^	22.41 ± 6.52^b,c^	2.62 (0.1217)	9.62 (0.0059)	36.98 (< 0.0001)
GST (μmol min^−1^ mg^−1^ protein)	28.06 ± 7.63^b^	12.18 ± 3.74^a,c,d^	22.81 ± 3.25^b^	22.14 ± 5.55^b^	12.30 (0.0024)	1.18 (0.2913)	14.58 (0.0012)
GSH (nmol GSH mg^−1^ of tissue)	5.59 ± 0.72^b^	2.29 ± 0.34^a,c^	5.74 ± 2.19^b^	4.16 ± 1.24	2.32 (0.1442)	3.20 (0.0894)	18.82 (0.0004)
SOD (units mg^−1^ protein)	16.28 ± 2.39^b^	10.19 ± 2.27^a,c,d^	14.47 ± 1.99^b^	16.32 ± 3.22^b^	14.22 (0.0013)	4.21 (0.0542)	4.05 (0.0585)
CAT (units mg^−1^ protein)	17.56 ± 9.07^b^	4.56 ± 2.10^a,c,d^	26.41 ± 7.80^b^	15.62 ± 3.25^b^	0.19 (0.6689)	15.22 (0.001)	21.73 (0.0002)
TBARS (nmol MDA mg^−1^ of protein)	2.37 ± 0.49^b^	4.20 ± 0.36^a,c,d^	02.02 ± 0.79^b^	2.48 ± 0.79^b^	6.41 (0.0204)	14.73 (0.0011)	17.94 (0.0004)
RS (% DCF of Arbitrary Unit)	100 ± 42.57^b^	146.83 ± 19.6^a,c,d^	101.34 ± 15.6^b^	100.64 ± 22.6^b^	4.73 (0.0424)	4.21 (0.0541)	4.46 (0.0482)
CP (nmol CP mg^−1^protein)	1.98 ± 0.66^b^	6.78 ± 1.51^a,c,d^	1.65 ± 0.64^b^	3.39 ± 0.90^b,c^	13.23 (0.0018)	19.64 (0.0003)	60.35 (< 0.0001)

*Note:* Data are presented as mean ± SD (*n* 5–6 rats per group) submitted to a two‐way ANOVA analysis of variance for analysis of *p* values of factors (*F*): Co (copaiba oil), MSG (obesity) and I (interaction). When *F* was significant, the means among groups were compared with Tukey's posttest (*p* < 0.05), with statistical differences represented by letters over numbers (a, CON_NS_; b, MSG_NS_; c, CON_Co_; d, MSG_Co_).

### 3.5 Effects of Co‐Resin Supplementation on Antioxidant Enzyme Activities and Oxidative Stress Markers in Plasma (Table 6) and Liver (Table 7) of Hypothalamic‐Obese (MSG) and Non‐obese (CON) Males

Neonatal treatment with MSG significantly influenced enzymatic activities of GR (*p* < 0.0001), GST (*p* = 0.002), GSH (*p* = 0.00061), and CAT (*p* < 0.0001) in the plasma of male rats. Thus, plasma from MSG_NS_ male rats presented lower enzymatic activities of GR (65.74%), GST (63.03%), GSH (57.00%), and CAT (82.63%) in comparison with the same enzymes in plasma of CON_NS_ animals. Moreover, MSG administration influenced plasma concentration of TBARS (*p* = 0.0251), RS (*p* = 0.0215), and CP (*p* = 0.0013) in males. As a consequence, the MSG_NS_ males group showed higher plasma levels of TBARS (71.50%), RS (41.89%), and CP (253.31%), compared with plasma of CON_NS_ males (Table 6).

**TABLE 6:** Effects of chronic Co supplementation on oxidative stress indicators for plasma of MSG‐obese and non‐obese (CON) males.

	Males (♂)				*F* (*p*)		
	CON_NS_	MSG_NS_	CON_Co_	MSG_Co_	I	Co	M
GPx (nmol NADPH min^−1^ mg^−1^ protein)	4.49 ± 2.57^b^	1.46 ± 0.24^a,d^	3.16b ± 1.87	4.21 ± 1.08^b^	9.24 (0.0065)	1.11 (0.3051)	2.16 (0.1568)
GR (nmol NADPH min^−1^ mgf^−1^ protein)	20.52 ± 6.1^b^	7.03 ± 2.44^a,c,d^	22.81 ± 8.05^b^	15.37 ± 3.1^b^	2.35 (0.1411)	7.27 (0.0139)	28.16 (< 0.0001)
GST (μmol min^−1^ mg^−1^ protein)	3.3 ± 1.78^b^	1.22 ± 0.36^a^	3.01 ± 1.59	1.67 ± 0.4	0.58 (0.4542)	0.027 (0.8704)	12.64 (0.002)
GSH (nmol GSH mg^−1^ of tissue)	17.63 ± 3.14^b^	7.58 ± 0.81^a,c,d^	15.54 ± 3.96^b^	14.11 ± 4.83^b^	9.43 (0.0060)	2.51 (0.1287)	16.71 (0.0006)
SOD (units mg^−1^ protein)	11.1 ± 1.03^b^	9.14 ± 0.47^a,d^	10.35 ± 0.59	11.18 ± 1.08^b^	16.84 (0.0006)	3.55 (0.0741)	2.74 (0.1134)
CAT (units mg^−1^ protein)	1451 ± 2.54^b^	2.52 ± 1.23^a,c,d^	15.25 ± 3.21^b^	10.47 ± 4.04^b^	9.40 (0.0061)	13.63 (0.0014)	50.70 (< 0.0001)
TBARS (nmol MDA mg^−1^ protein)	4.14 ± 1.18^b^	7.1 ± 1.36^a,c,d^	4.76 ± 0.64^b^	4.04 ± 1.04^b^	15.86 (0.0007)	6.98 (0.0156)	5.860 (0.0251)
RS (% DCF of Arbitrary Unit)	100 ± 18.87^b^	141.89 ± 27.13^a,c,d^	91.99 ± 15.14^b^	89.96 ± 9.36^b^	7.56 (0.0124)	14.07 (0.0013)	6.22 (0.0215)
CP (nmol CP mg^−1^protein)	2.57 ± 1.05^b^	9.08 ± 3.73^a,c,d^	2.56 ± 1.04^b^	2.96 ± 1.3^b^	10.86 (0.0036)	10.92 (0.0035)	13.85 (0.0013)

*Note:* Data are presented as mean ± SD (*n* 5–7 rats per group) submitted to a two‐way ANOVA analysis of variance for analysis of *p* values of factors (*F*): Co (copaiba oil), MSG (obesity) and I (interaction). When *F* was significant, the means among groups were compared with Tukey's posttest (*p* < 0.05), with statistical differences represented by letters over numbers (a, CON_NS_; b, MSG_NS_; c, CON_Co_; d, MSG_Co_).

Co‐resin supplementation significantly modulated plasma enzyme activities of GR (*p* < 0.0001) and CAT (*p* < 0.0001) in males. Moreover, I (Co × M) effects were also observed for plasma activities of GPx (*p* = 0.0065), GSH (*p* = 0.0060), SOD (*p* = 0.0006), and CAT (*p* = 0.0061). Thus, activities of the GPx (65.32%), GR (54.26%), GSH (46.28%), SOD (18.25%), and CAT (5.93%) were significantly elevated in the MSG_Co_ supplemented males, in comparison with MSG_NS_ males. Effects of Co‐resin supplementation were also observed for plasma levels of TBARS (*p* = 0.0156), RS (*p* = 0.0013), and CP (*p* = 0.0035), with MSG_Co_ males presenting reduced plasma levels of TBARS (43.10%), RS (36.60%), and CP (67.40%) in plasma, when compared with MSG_NS_ males (Table 6).

Neonatal MSG treatment also influenced antioxidant enzyme activity in the liver of male rats with alterations in GPx (*p* = 0.0074), GR (*p* < 0.0001), GST (*p* < 0.0001), and CAT (*p* < 0.0001) enzyme activities. As such, the MSG_NS_ male group demonstrated reductions in hepatic enzyme activities of GPx (78.66%), GR (74.91%), GST (63.59%), and CAT (64.40%) in relation to CON_NS_ male rats. Moreover, MSG administration influenced TBARS (*p* = 0.0156) and RS (*p* = 0.0013) in male livers, with elevated hepatic values of TBARS (213.53%) and RS (32.80%) found in MSG_NS_ males in comparison with the CON_NS_ male group.

In males, Co‐resin supplementation modified enzyme activities of the GST (*p* < 0.0001) and CAT (*p* = 0.0021) in hepatic tissue. Moreover, we also observed an I (Co × M) effect on all hepatic enzyme activities evaluated [GPx (*p* = 0.0355), GR (*p* = 0.0083), GST (*p* = 0.0002), GSH (*p* = 0.0141), SOD (*p* = 0.0439), and CAT (*p* = 0.0463)]. Thus, MSG_Co_‐supplemented males presented augmented activities of GPx (72.48%), GR (54.58%), GST (60.67%), GSH (47.62%), SOD (21.74%), and CAT (58.28%) in relation to the same hepatic enzymes in MSG_NS_ males.

The Co‐resin supplementation influenced TBARS (*p* = 0.0045) and RS (*p* = 0.0088) in the liver of males. Here, we also observed I (Co × M) effects on TBARS (*p* = 0.0018), RS (*p* = 0.0165), and CP (*p* = 0.0151) levels. As a consequence, MSG_Co_‐supplemented male rats presented decreased values of TBARS (47.70%), RS (46.03%), and CP levels (39.14%), when compared with the MSG_NS_ male group (Table 7).

**TABLE 7:** Effect of chronic supplementation with Co on oxidative stress indicators for liver in MSG‐obese and non‐obese males.

	Males (♂)				*F* (*p*)		
	CON_NS_	MSG_NS_	CON_Co_	MSG_Co_	I	Co	M
GPx (nmol NADPH min^−1^ mg^−1^ protein)	35.34 ± 19.04^b^	7.54 ± 4.36^a,c,d^	31.62 ± 17.49^b^	27.76 ± 6.1^b^	5.08 (0.0355)	2.42 (0.1356)	8.89 (0.0074)
GR (nmol NADPH min^−1^ mgf^−1^ protein)	45.83 ± 8.4^b,d^	11.5 ± 3.91^a,c,d^	41.56 ± 12.61^b,d^	25.32 ± 3.12^a,b,c^	8.75 (0.0083)	2.39 (0.1375)	66.97 (< 0.0001)
GST (μmol min^−1^ mg^−1^ protein)	27.93 ± 2.06^b^	10.17 ± 3.47^a,c.d^	30.5 ± 4.41^b^	25.86 ± 3.58^b^	21.67 (0.0002)	41.89 (< 0.0001)	63.05 (< 0.0001)
GSH (nmol GSH mg^−1^ of tissue)	10.47 ± 3.83^b^	5.52 ± 1.45^a,d^	9.49 ± 2.48	10.54 ± 2.7^b^	7.23 (0.0141)	3.29 (0.0847)	3.05 (0.0962)
SOD (units mg^−1^ protein)	17.32 ± 2.94^b^	13.64 ± 0.59^a^	17.01 ± 3.4	17.43 ± 1.82^b^	4.63 (0.0439)	3.31 (0.0838)	2.91 (0.1036)
CAT (units mg^−1^ protein)	15.14 ± 4.22^b^	5.39 ± 1.57^a,c,d^	17.02 ± 2.14^b^	12.92 ± 4.18^b^	4.51 (0.0463)	12.49 (0.0021)	27.12 (< 0.0001)
TBARS (nmol MDA. mg^−1^ protein)	2.48 ± 0.75^b^	5.43 ± 0.85^a,c,d^	2.63 ± 1.33^b^	2.84 ± 0.78^b^	12.89 (0.0018)	10.26 (0.0045)	17.03 (0.0005)
RS (% DCF of Arbitrary Unit)	96.76 ± 23.14^b^	143.99 ± 32.94^a,c,d^	93.33 ± 31.32^b^	77.72 ± 28.07^b^	6.85 (0.0165)	8.42 (0.0088)	1.73 (0.2029)
CP (nmol CP mg^−1^protein)	2.07 ± 0.37^b^	6.49^b^ ± 2.47^a,c,d^	2.64 ± 0.47^b^	3.95 ± 0.67^b^	7.07 (0.0151)	2.85 (0.1067)	24.10 (< 0.0001)

*Note:* Data are presented as mean ± SD (*n* 5–7 rats per group) submitted to a two‐way ANOVA analysis of variance for analysis of *p* values of factors (*F*): Co (copaiba oil), MSG (obesity) and I (interaction). When *F* was significant, the means among groups were compared with Tukey's posttest (*p* < 0.05), with statistical differences represented by letters over numbers (a, CON_NS_; b, MSG_NS_; c, CON_Co_; d, SG_Co_).

### 3.6 Western Blotting Analyses of Plasma (Figure 2A,C) and Hepatic (Figure 2B,D) SOD and CAT Protein Levels in Hypothalamic‐Obese (MSG) and Non‐obese (CON) Females

**FIGURE 2:**
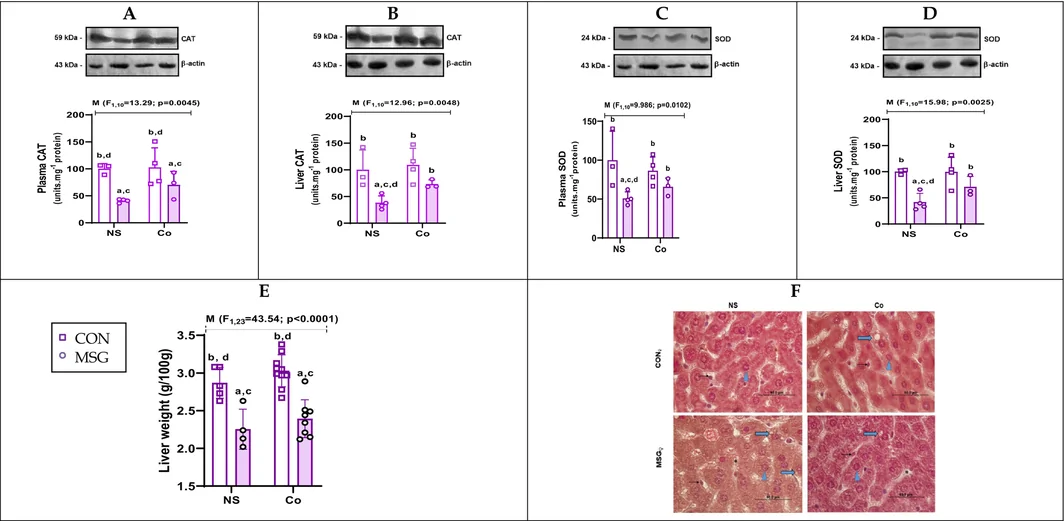
Effects of copaiba oil supplementation on antioxidant enzyme activity and liver morphology in MSG‐obese and non‐obese females supplemented with Co. (A–D) Protein activities of SOD and CAT enzymes in plasma and liver. Data are presented as mean ± SD (*n* = 3–4 rats per group) submitted to a two‐way ANOVA analysis of variance for analysis of *p* values of factors (F): Co (copaiba oil), MSG (obesity), and I (interaction). When *F* was significant, the means among groups were compared with Tukey's posttest (*p* < 0.05), with statistical differences represented by letters over numbers (a‐CON_NS_; b‐MSG_NS_; c‐CON_Co_; d‐MSG_Co_). (A) CAT plasma; (B) CAT liver; (C) plasma SOD; (D) Liver SOD. (E) Liver weight. (F) Histological analysis of liver tissue, HE staining. (*n* 4–10 rats per group). Representative photomicrographs of hepatocytes with Hematoxylin and Eosin (HE) staining, 100× transverse section magnification. The following are indicated: Nuclei (triangle); sinusoidal capillaries (asterisk); Kupffer cells (thin black arrow) hepatocytes with fat microvesicles (blue thick arrow). CAT, catalase; Co, supplemented with Co‐resin; CON, non‐obese; MSG, obese; NS, non‐supplemented; SOD, superoxide dismutase.

The MSG‐treatment significant have changed plasmatic (CAT: *p* = 0.0045 and SOD: *p* = 0.0102) and hepatic (CAT: *p* = 0.0048 and SOD: *p* = 0.0025) antioxidant protein levels. Thus, lower CAT (59.22%) and SOD (49.14%) protein contents were observed in plasma of MSG_NS_ female, in relation to the plasma of the CON_NS_ female animals. Similarly, lower protein concentrations of CAT (61.85%) and SOD (58.32%) were found into liver of MSG_NS_ females in relation to the livers of CON_NS_ females. Although Co‐resin nonsignificant have changed plasmatic and hepatic protein levels of CAT and SOD, it was possible to observe that females in MSG_Co_ group showed values statistically similar those found in the CON_NS_ groups (Figure 2A,B).

### 3.7 Western Blotting Analyses of Plasmatic (Figure 3A,C) and Hepatic (Figure 3B,D) SOD and CAT Protein Levels in Hypothalamic‐Obese (MSG) and Non‐obese (CON) Males

**FIGURE 3:**
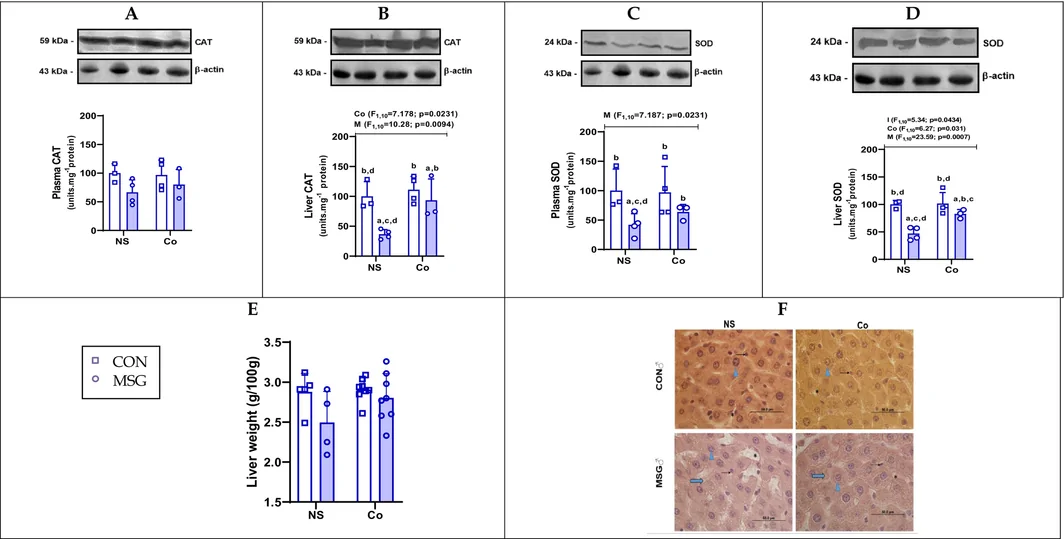
Effects of Co‐resin supplementation on antioxidant enzyme activity and liver morphology in MSG‐obese and non‐obese males supplemented with Co. (A–D) Protein activities of SOD and CAT enzymes in plasma and liver. Data are presented as mean ± SD (*n* = 5–7 rats per group) submitted to a two‐way ANOVA analysis of variance for analysis of *p* values of factors (*F*): Co (copaiba oil), MSG (obesity), and *I* (interaction). When *F* was significant, the means among groups were compared with Tukey's posttest (*p* < 0.05), with statistical differences represented by letters over numbers (a‐CON_NS_; b‐MSG_NS_; c‐CON_Co_; d‐MSG_Co_). (A) CAT plasma; (B) CAT liver; (C) plasma SOD; (D) liver SOD. (E) Liver weight. (F) Histological analysis of liver tissue, HE staining. (*n* = 4–10 rats per group). Representative photomicrographs of hepatocytes with Hematoxylin and Eosin (HE) staining, 100× transverse section magnification. The following are indicated: Nuclei (triangle); sinusoidal capillaries (asterisk); Kupffer cells (thin black arrow); hepatocytes with fat microvesicles (blue thick arrow). Data presented as percentage (%). Legend: CON: Non‐obese; MSG: Obese; NS: Non‐supplemented; Co: Supplemented with Co‐resin; Catalase (CAT); Superoxide Dismutase (SOD).

Neither MSG treatment nor Co‐resin supplementation influenced CAT or SOD protein levels in males plasma (Figure 3A,C). In contrast, significant effects of MSG were observed in males liver regarding protein levels of CAT (*p* = 0.009) and SOD (*p* = 0.0007). Thus, in MSG_NS_ group, a lower CAT (63.49%) and SOD (52.90%) protein immuno‐content were observed in the hepatic tissue, when compared with CON_NS_ group (Figure 3B,D). Moreover, in males, Co‐resin supplementation also modulated hepatic CAT (*p* = 0.023) and SOD (*p* = 0.031) protein levels. Moreover, into liver also were found I (Co × MSG) effects to SOD (*p* = 0.0434). Thus, MSG_Co_ groups demonstrated increases of 67.08% and 46.44%, respectively, for hepatic CAT and SOD levels in relation to MSG_NS_ males.

### 3.8 PCA for Plasma and Hepatic Stress Biomarkers and WB Analysis of SOD and CAT (Figures 4 and 5)

**FIGURE 4:**
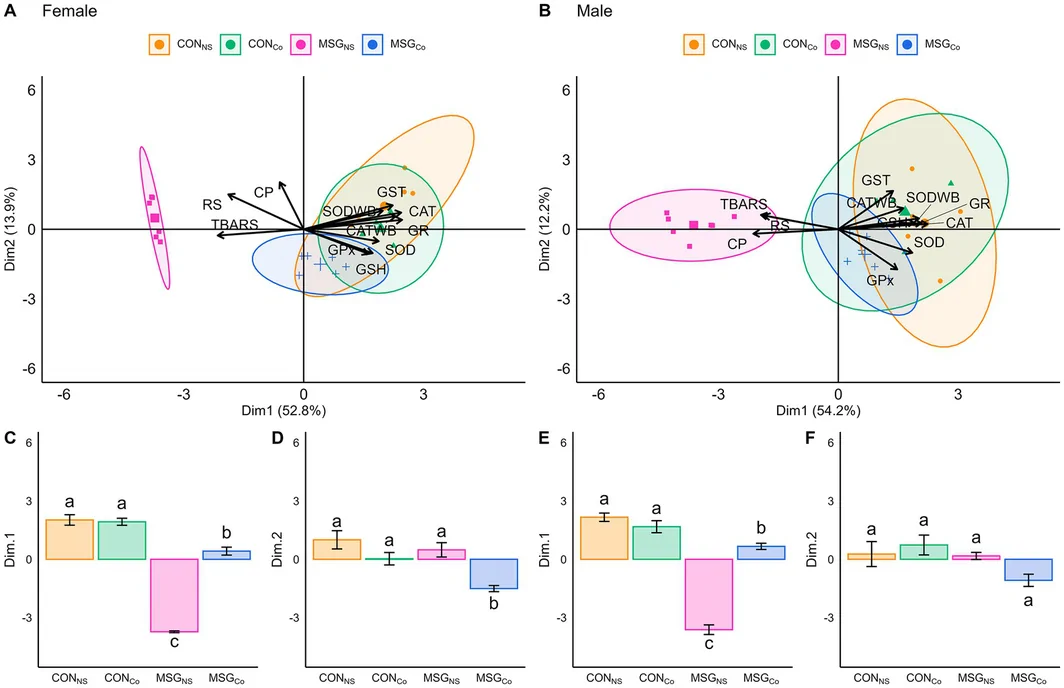
Principal component analysis (PCA) of plasma oxidative biomarkers in female (A, C, D) and male (B, E, F) non‐obese (CON) and obese (MSG) rats. Upper panels represent the ordering diagrams of eigenvalues and eigenvectors, with ellipses separating the groups (A—females; and B—males). The lower panels show means ± SD of the dimension (Dim) 1 (C, E—females) and Dim 2 (D, F—males). Distinct letters on the bars in the lower panel graphs indicate statistically significant differences among groups, as per the Tukey test (*p* < 0.05) for multiple comparisons. CON_Co_‐♀, on‐obese supplemented with Co‐resin females; CON_Co_‐♂, on‐obese supplemented with Co‐resin males; CON_NS_‐♂, on‐obese non‐supplemented males; MSG_Co_‐♀, bese supplemented with Co‐resin females; MSG_Co_‐♂, bese supplemented with Co‐resin males; MSG_NS_‐♀, bese non‐supplemented females; MSG_NS_‐♂, Bese non‐supplemented males.

**FIGURE 5:**
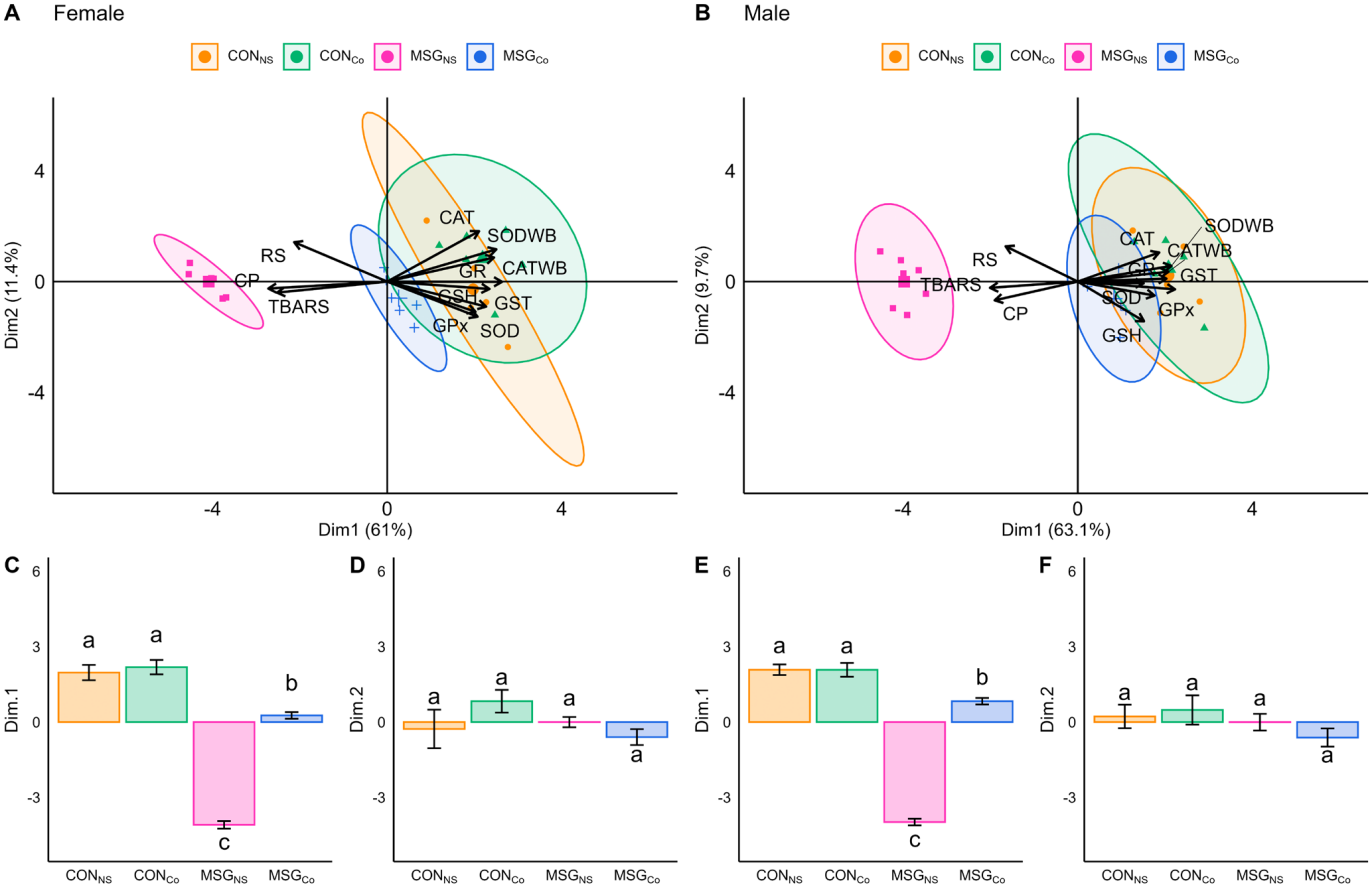
Principal component analysis (PCA) of hepatic oxidative biomarkers in female (A, C, D) and male (B, E, F) non‐obese (CON) and obese (MSG) rats. Upper panels represent the ordering diagrams of eigenvalues and eigenvectors, with ellipses separating the groups: (A) females; and (B) males. The lower panels show means ± SD of the *Dim* 1: (C, E) females; and *Dim* 2: (D, F) males. Distinct letters on the bars in the lower panel graphs indicate statistically significant differences among groups, as per the Tukey test (*p* < 0.05) for multiple comparisons. CON_Co_‐♀, non‐obese supplemented with Co‐resin females; CON_Co_‐♂, non‐obese supplemented with Co‐resin males; CON_NS_‐♂, non‐obese non‐supplemented males; MSG_CO_‐♀, obese supplemented with Co‐resin females; MSG_Co_‐♂, obese non‐supplemented males; MSG_NS_‐♀, obese non‐supplemented females; MSG_NS_‐♂, obese non‐supplemented males.

The contrasting plasma and hepatic oxidative stress profiles of female and male MSG and CON rats, as well as the effects of Co‐resin supplementation are clearly observed in PCA analyses, shown in Figures 4 and 5.

In plasma, the set of analyzed variables explained 66.46% and 66.69% of variability, respectively in female and male rats. In females, the Dim1 represents 52.77% (auto‐value = 5.80) of this variability, which originates from GR (13.8%), CAT (13.6%), CATWB (12.1%), GST (11.3%), TBARS (10.5%), and SODWB (8.93%). For males, Dim1 explains 54.23% of this variability, which is distributed as CAT (12.8%), CP (11.6%), GR (11.2%), SODWB (10.5%), TBARS (9.65%), GSH (9.28%), and RS (8.9%). Thus, in CON_NS_ and CON_Co_ female groups, positive scores for antioxidants are found, indicating higher immunoreactivity in relation to obese females (MSG_NS_). In contrast, pro‐oxidative markers such as TBARS, RS, and CP were more elevated in MSG_NS_ females, in comparison with the other groups. This effect is corrected by Co‐resin supplementation, with values of MSG_Co_ females to bring those of CON groups closer. Thus, significant MSG (*p* < 0.001), Co (*p* < 0.001), and interaction (*p* < 0.001) effects were observed, as shown in Figure 4C,D. A similar profile may be observed in MSG_NS_ males, which showed higher TBARS, RS, and CP, and lower antioxidant enzyme activity in relation to CON_NS_ males, with Co‐resin supplementation to correct this pro‐oxidative state. Therefore, significant effects of MSG (*p* < 0.001), Co (*p* < 0.001), and interactions (*p* < 0.001) were confirmed (Figure 4E,F).

Dim2 contributed 13.92% (auto‐value = 1.53) and 12.23% (auto‐value = 1.35) respectively to variability of data. In females plasma, Dim2 was determined by variables RS (19.5%), GSH (9.16%), GPx (9.67%) and CP (35.5%). While in males, this dimension was constituted by GST (31.1%), GPx (34.1%), SOD (11.7%) and CATWB (9.44%). Thus, female MSG_NS_ animals showed high values of RS and CP in relation to other groups. However, in Co‐resin supplemented MSG and CON females, GPx and GSH presented negative scores, suggesting a reduction in plasma oxidative stress. These results are confirmed by the significant effects of MSG (*p* < 0.01), Co (*p* < 0.001) and interactions (*p* = 0.177), as shown in Figure 4A. In contrast, despite demonstrating a similar profile for Co‐resin supplementation modulation of plasma oxidative status in males, no significant statistical difference was observed. In this regard, no significant effects of MSG (*p* = 0.05); Co (*p* = 0.23) or interactions (*p* = 0.06) were found for males, as shown in Figure 4B.

In the liver, variables grouped in Dim1 and Dim2 explain 72.76% (females) and 72.34% (males) of data variability, with Dim1, which contributed to a greater part of this effect in females (60.96%; auto‐value =6.71) and males (63.06%; auto‐value = 6.94). In females, Dim1 was constituted of variables CP (12.2%), CATWB (11.4%), TBARS (10.5%), GR (9.83%), GSH (9.01%) and GST (8.43%), while in the males it was constituted of variable GST (12.5%), SODWB (11.7%), CATWB (10.9%), GR (10.4%), TBARS (10.2%), CP (9.16%), GPx (7.81%) and SOD (5.75%). Thus, in Dim1, TBARS and CP are higher, while CATWB, GR, GSH and GST are lower in MSG‐_NS_ females, in comparison with non‐obese (CON) females. These hepatic pro‐oxidative profiles were attenuated by Co‐resin supplementation, as shown in Figure 5, and showed significant effects of MSG (*p* < 0.001), Co (*p* < 0.001) and interactions (*p* < 0.001).

In the males, Dim1 showed higher levels of TBARS and CP, followed by lower presence of GST, SODWB, CATWB, GR, GPx, and SOD in MSG_NS_ rats when compared with the other experimental groups. This effect was modified by Co‐resin supplementation. Thus, significant effects of MSG (*p* < 0.001), Co (*p* < 0.001), and interaction (*p* < 0.001) are observed in Figure 5E,F.

Dim2 contributed to 11.37% of variability in females regarding liver (auto‐value = 1.25) and 9.70% of variability in males (auto‐value = 1.07). For females, Dim2 is constituted of variables such as SODWB (12%), RS (17.8%), CAT (29.2%), SOD (14.2%) and GPx (10.5%), while in males, it is composed of CAT (18.1%), RS (26.7%) and GSH (33.7%).

Despite observations of higher SODWB, CAT, SOD, and GPx and lower RS values in CON_NS_ and CON_Co_ females, these effects were not statistically significant, as shown in Figure 5 (MSG: *p* = 0.18; Co: *p* = 0.64; interactions: *p* = 0.08). Similarly, for Dim2, for liver oxidative status analysis, no statistical effects of MSG (*p* = 0.159), Co (*p* = 0.624), and interactions (*p* = 0.324) were found.

### 3.9 Histomorphometric Analysis of Livers From Hypothalamic‐Obese (MSG) and Non‐obese (CON) Females (Figure 2F)

In non‐obese female groups (CON_NS_ and CON_Co_), it was observed normal liver tissue organization, with aggregates of liver cells (hepatocytes) surrounded by fenestrated endothelial cells forming sinusoidal capillaries. These structures are populated by resident macrophages, Kupffer cells (KC). Hepatocytes exhibited well‐defined cytoplasm, nuclei, and nucleoli (Figure 2F and Table S1). MSG treatment significantly influenced liver weight (Figure 2E) in females (*p* < 0.0001), with a 24.31% reduction in liver weight in the MSG_NS_ group when compared with the CON_NS_ group. However, the number of KC and nuclei in females' livers was not significantly affected by MSG treatment or Co‐resin supplementation.

Despite this, in the MSG_NS_ female group, histomorphology changes were observed, such as dilated sinusoidal capillaries, peripherally‐located nuclei, tinctorial alterations in cytoplasm, and the presence of lipid microvesicles. These alterations were restored in livers from the MSG_Co_ female group and indicated a potential protective effect of Co‐resin supplementation into hepatic tissue (Figure 2F).

Regarding hepatic lipid deposition, females from MSG_NS_ and MSG_Co_ groups presented Grade 1 hepatic steatosis, demonstrating 41% and 36% of fat microvesicles, respectively. Although both groups shared the same steatosis grade, Co‐resin supplementation reduced lipid microvesicles percentage. Interestingly, even the CON_Co_ group exhibited Grade 1 steatosis (34%), suggesting some presence of lipid accumulation in this group as well. Neither MSG nor Co‐resin supplementation significantly changed KC and nuclei numbers in livers from MSG and CON females (Table S1).

### 3.10 Histomorphometric Analysis of Livers From Hypothalamic‐Obese (MSG) and Non‐obese (CON) Males (Figure 3F)

In the non‐obese male groups (CON_NS_ and CON_Co_), liver tissue displayed normal organization, with hepatocyte aggregates surrounded by sinusoidal capillaries and KC. Hepatocytes had clearly defined cytoplasm, nuclei, and nucleoli (Figure 3F).

Unlike females, none of the hepatic parameters, including liver weight (Figure 3E), were significantly changed in males by either MSG‐induced obesity or Co‐resin supplementation. However, in the MSG_NS_ male group, changes in liver tissue morphology were evident, similarly to observations in females. These included dilated sinusoidal capillaries, nuclei positioned at the cell periphery, cytoplasmic staining differences, and presence of lipid microvesicles. These changes were also reversed in the MSG_Co_ male group, as shown in Figure 3F.

Regarding hepatic steatosis, only the MSG_NS_ male group presented grade 1 steatosis (34%). The other male groups exhibited only the normal presence of fat microvesicles, which was not enough to be classified as hepatic steatosis. Neither MSG nor Co‐resin supplementation significantly changed KC and nuclei numbers in the liver from MSG and CON males (Table S2).

## 4 Discussion

High doses of MSG, administered during the first PND, induce damage in the hypothalamic nucleus, particularly destroying the hypothalamic ARC. Here, we confirmed that MSG‐hypothalamic injured female and male rodents developed high adiposity, hyperglycemia, hypertriglyceridemia, and IR in adulthood. The excessive adiposity and metabolic abnormalities found in MSG‐obese rodents have been attributed to autonomic dysfunctions, hyperinsulinemia, and excessive cortisol since this obese model does not develop hyperphagia (Macho et al. 2000; Araujo et al. 2017). Interestingly, MSG‐hypothalamic obese rats show a combination of high body fat and low muscle mass, simulating the sarcopenic state that is also presented by obese human individuals (Zazula et al. 2023; Habboub et al. 2025). Moreover, modified hypothalamic gonadal axis with an exacerbated reduction in sexual hormones and infertility are commonly found in MSG‐treated rats and differentially affect females' and males' metabolism (Quines et al. 2019), including those aspects involving oxidative stress (Rudyk et al. 2018). Thus, in the present study, we evaluated the influence of Co‐resin supplementation on plasma and liver oxidative stress, considering the sex influence in non‐obese and MSG‐hypothalamic obese models.

Co‐resin contains several compounds with anti‐inflammatory properties. WAT expansion is intimately associated with a pro‐inflammatory state. However, Co‐resin supplementation effects on WAT are scarce and have presented contradictory results. In this regard, Co‐resin supplementation has not influenced the WAT of non‐obese rats (healthy) (Rocha et al. 2022) but showed a positive impact on WAT from a high sucrose‐diet obese model (Telles et al. 2022).

We recently published data showing that Co‐resin supplementation did not change WAT content or adipocyte histology in MSG‐treated males, but caused a slight reduction in WAT and increased adipocyte number in MSG‐obese females, indicating sex influences on Co‐resin supplementation effects (Kailer et al. 2024). In agreement, here, Co‐resin supplementation reduced TGL levels in MSG‐females, but worsens TGL profile in MSG‐obese males. Tabassum et al. (2022) demonstrated that TGL levels are high in men, when compared with women's answers. These sex effects on lipids have also been observed in obese adolescents and may be related to differences in WAT distributions or IR (Calcaterra et al. 2021).

Liver metabolism is essential to preserve energy homeostasis. Deleterious hepatic effects are common in obesity. Thus, rodents' liver or obese humans frequently develop excessive lipid deposition and hepatic inflammation, which are intimately associated with increased oxidative stress. Moreover, oxidative stress in liver is also influenced by sex (Bautista et al. 2019; Rezzani and Franco 2021; Sayaf et al. 2022).

Neonatal MSG treatment causes liver abnormalities and increases lipid deposition in hepatocytes in these rodents (Nabi et al. 2022). In the present study, we demonstrated that MSG‐treated female and male rats showed morphological changes in their hepatic tissues, particularly dilated sinusoid capillaries, decentralized nuclei, and augmented microvesicular lipid deposition without increasing KC numbers. In addition, we also showed herein that livers of MSG‐obese female and male animals did not present typical NASH characteristics. These data confirm other studies showing that MSG‐obese mice present normal tumor necrosis factor (TNF‐α) expression into KC (Tsuneyama et al. 2011), as well as develop simple steatosis with no progression to more severe forms of the disease.

It is well established that oxidative stress has a significant role in the pathophysiology of metabolic diseases, such as diabetes. In this regard, hepatic and plasma activity of antioxidant enzymes was significantly lower in diabetic than in healthy individuals (Rocha et al. 2022). In agreement, we demonstrated herein that MSG‐treated females and males show reduced plasmatic and hepatic activities of antioxidant enzymes (GPx; GR; GST; GSH; SOD; and CAT). Moreover, we also showed that MSG‐obese female and male rats present higher levels of plasma and hepatic stress markers (TBARS, RS, and CP content). Hepatic enzyme activity dysfunctions have been observed in MSG‐obese model (Ayala et al. 2014), with reductions reported in GSH, SOD, and CAT levels when compared with non‐obese animals. Accordingly, we found reduced SOD and CAT protein immunoblotting contents in livers of female and male MSG‐obese rats.

Augmented lipid depositions in hepatocytes have been associated with increased ROS production (Ozougwu 2017), a mediator of damage to cell structures, such as lipids, membranes and proteins leading to deleterious effects in several signaling pathways, including hepatic insulin responsiveness (Del Campo et al. 2023). Thus, excessive hepatic ROS and high fat liver deposition have a close relationship with IR. In agreement, here we confirmed the presence of IR in MSG‐obese rats with mild hepatic fat accumulation. So, it was suggested that an early interrelationship among hepatic lipid metabolism and ROS in rodents' livers, treated with MSG. Mitochondria are an important source of ROS in the liver, and mitochondrial dysfunctions with excessive ROS generation are found in obese patients (Mello et al. 2018). Neonatal administration of MSG induces mitochondrial dysfunction, oxidative stress, and hepatotoxicity. Analysis of mitochondrial function in the liver of MSG‐obese rats showed higher fatty acid oxidation capacity (Elkattawy et al. 2023). Moreover, Yamazaki et al. (2009) proposed that the increase in TGL accumulation in livers of MSG mice results from an increased lipid synthesis and/or free fatty acid influx to the liver. A combined analysis of these data reinforced the importance of lipids metabolism to hepatic oxidative stress in MSG‐obese rats. For this reason, antioxidant supplementation could be an important approach to avoid deleterious effects of ROS in several tissues and preserve oxidative balance and health.

In this regard, natural compounds seem to be able to restore oxidative system imbalances commonly found in MSG‐obese model. For example, ethanolic extract from *Coccinia grandis* leaves showed natural antioxidant and free‐radical scavenging properties, as well as anti‐inflammatory and anti‐apoptotic effects that helped restore oxidative system and metabolic dysfunction in MSG‐obese fed a high fat diet (Banerjee et al. 2020). The oral Co‐resin supplementation to healthy male Wistar rats exerts intense antioxidant and anti‐inflammatory actions during skin ischemia and reperfusion by regulating GSH and TBARS levels (de Lima Silva et al. 2009). Moreover, oral Co‐resin administration was able to reduce activity of myeloperoxidase, a biochemical marker of neutrophil infiltration, in the intestinal model of acetic acid‐induced colitis (Assis et al. 2014). To the best of our knowledge, there is no study evaluating the effects of Co‐resin oral supplementation on the oxidative system of obese rodents, in special evaluating sexual dimorphism influences.

The Co‐resin used in the present study presented good antioxidant activity, as shown by our analysis of ABTS and DPPH. Thus, our study showed, for the first time, that Co‐resin supplementation was able to prevent hepatic and plasma imbalances in the antioxidant system in MSG‐obese female and male animals. Moreover, we also demonstrated that Co‐resin supplementation significantly reduced levels of stress markers, such as TBARS, RS, and CP content in the plasma and liver of both female and male MSG‐obese groups.

Co‐resin has shown similar hepatoprotective effects in non‐obese rodents. For example, in healthy male Wistar rats, the administration of Co‐resin prophylactically for 7 days and therapeutically (at 0.63 mL kg^−1^) at 2 h after acute acetaminophen intoxication promoted hepatoprotection against paracetamol‐induced hepatic damage (Teixeira et al. 2013). Similarly, Co‐resin supplementation (at 0.58 mg kg^−1^) improves hepatic lesions induced by arthritis in male Holtzman rats (Gomes et al. 2018). According to these authors, the hepato‐protective effects of Co‐resin supplementation could be attributed primarily to its antioxidative actions.

β‐caryophyllene is reported to be the major anti‐oxidative agent present in Co‐resin. A study by Ames‐Sibin et al. (2018) showed that isolated β‐caryophyllene supplementation, at the dose of 430 mg kg^−1^, abolished the increase in protein CP groups and myeloperoxidase expression in the liver and plasma of arthritic rats. Moreover, at a smaller dose, β‐caryophyllene abrogated increased levels of ROS and reduced glutathione in the arthritic liver.

Interestingly, antioxidant supplementation with Co‐resin and β‐caryophyllene does not apparently modify mitochondrial function (Ames‐Sibin et al. 2018). This suggested that their effects are mediated by the endogenous antioxidant system during pathological oxidative states. These effects were clearly shown in the current study, where Co‐resin supplementation restored plasma and hepatic antioxidant systems and reduced the production of stress markers in the hypothalamic MSG‐obese model.

Moreover, we also observed that Co‐resin supplementation may increase plasma and hepatic activities of SOD and CAT in MSG‐obese rats, indicating direct regulatory effects on these enzymes. Essential Co‐oil is a concentrated liquid obtained by steam distillation of Co‐resin and a recent in vitro study confirmed the greater potential antioxidant and anti‐inflammatory properties of essential oils obtained from several copaiba species (Cebollada et al. 2023). In the current study, Co‐resin was extracted from *C. officinalis*, which presents 63.63% of sesquiterpenes and 36.38% diterpenes in its composition, where the major sesquiterpenes are β‐caryophyllene (De Galúcio et al. 2016). β‐caryophyllene has been reported to induce the expression of nuclear translocation of nuclear factor erythroid 2‐related factor, which upregulates antioxidant defense genes and improves the cellular GSH antioxidant system in C6 glioma cells in rats with cerebral ischemia‐reperfusion injury (Assis et al. 2014). Thus, it is possible that this compound can regulate SOD and CAT activities and content in MSG‐obese rats.

As previously mentioned, Co‐resin and essential oils showed anti‐inflammatory properties that could also contribute to plasma and hepatic antioxidant systems observed in this study. In this regard, several in vitro and animal model studies have demonstrated that Co‐resin or essential oil, as well as its isolated compounds, are able to reduce the expression of cyclooxygenase 2 and several pro‐inflammatory cytokines, such as TNF‐α, interleukin‐6, and interleukin‐1β (Campos et al. 2021). Moreover, β‐caryophyllene is reported to inhibit activation of nuclear factor kappa light chain enhancer of B cells, a key transcriptional element that controls several cytokines' expressions (da Trindade et al. 2018; Assis et al. 2014). Recently, a study carried out by Cebollada et al. (2023) showed that essential Co extracted from *C. officinalis* has anti‐inflammatory effects that are independent from lipid peroxidation, 5‐lipoxygenase inhibition or antioxidant effects. In this sense, anti‐inflammatory effects of essential Co extracted from *C. officinalis* could be mediated by the action on cannabinoid receptor type 2 (Ferro et al. 2018).

It is interesting to note that Co essential oil upregulates the mitogen‐activated protein kinase and Janus kinase/STAT signaling pathways in HepG2 hepatocyte cell line (Urasaki and Le 2019). In contrast, β‐caryophyllene negatively regulated the phosphoinositide 3‐kinase/protein kinase B/mTOR signaling pathway in a dose‐dependent manner in HepG2. According to the authors, these responses indicated a collective behavior of Co essential oil that results from the complex interactions of its many constituents, where this synergy is critical to the biological effects of oil (Cardinelli et al. 2023). Thus, the beneficial effects of Co‐resin supplementation, obtained here, may have both antioxidant and anti‐inflammatory properties, although these last ones were not evaluated here.

Sex affected the antioxidant system, whose males have frequently shown more oxidative stress than females, as it is demonstrated in several species including rats and humans (Martínez de Toda et al. 2023). A recent review clearly demonstrates that the liver is a sexually dimorphic organ and that prevalence, progression, and severity of multiple liver diseases, including MASLD, differ between men and women (Bellanti et al. 2013). This review also emphasizes that the impact of sex research remains an under‐characterized condition in the liver. As such, several studies have found greater antioxidant potential in females than in males (Kočar et al. 2026), a response that is related to the estrogen‐linked antioxidant advantage (Di Franco et al. 2022).

Moreover, in rodents, it has been demonstrated that endoplasmic reticulum stress, a mechanism that generates ROS, is augmented by testosterone (Rossetti et al. 2019). As aforementioned, due to ARC lesions, MSG‐obese rats show reduced sexual hormone circulating levels (Yamazaki et al. 2009). However, the obtained data suggested that sexual dimorphism is persistent in MSG‐neonatally treated rodents (Hernández‐Bautista et al. 2014). A study by Altintas et al. (2025) of MSG‐obese females showed a higher adiposity index and worse genotoxicity effects. We previously demonstrated that MSG‐obese females show altered cholinergic neuronal activity when compared with MSG‐obese males (Grassiolli et al. 2006). Prophylactic supplementation with multi‐probiotics in MSG‐administered rats during their early lives prevents obesity development in female animals and strongly abrogates but does not prevent obesity in male animals (Rudyk et al. 2018). Accordingly, in the current study, Co‐resin supplementation of MSG‐obese females demonstrated greater beneficial effects on liver histology and on oxidative stress system than MSG‐obese males.

Sexual liver dimorphism also seemed to be influenced by growth hormone (GH) profile, which can modulate histology and hepatic gene expression differently in male and female rodents (Matz‐Soja et al. 2025). MSG‐neonatal treated rats showed lower GH levels with sexual dimorphism that impacted this profile. Thus, according to the study of (Maiter et al. 1991), MSG‐administered females, but not males, in the prepubertal phase have diminished GH circulating levels. In addition, MSG‐obese females in adult life were more responsive to human GH‐releasing factor than MSG‐obese males (Wakabayashi et al. 1986). Notably, in that study, Co‐resin was administered throughout life. Further studies about the impact of Co‐resin supplementation on the GH axis should be carried out.

Finally, as it has been described here, fasting values of TGL, glucose and TyG index were improved by Co‐resin supplementation in MSG‐obese females, but this answer did not happen in MSG‐obese male rats. Co‐resin probably acted as a direct antioxidant scavenger in both sexes; however, in MSG females, it also improved metabolism, including insulin responsiveness and TGL levels. Therefore, MSG‐females Co‐resin supplementation reduced liver overload as well as decreased ROS generation. Consequently, there was a more beneficial health impact on those studied rodent lives.

### 4.1 Limitations and Future Perspectives

This study has potential limitations.

First, a precise analysis of compounds concerning the Co‐resin used in this trial was not carried out. However, according to at least three important reviews (Valenzuela et al. 2026; da Trindade et al. 2018; Cardinelli et al. 2023), the main constituents found in this oil are similar and their known effects were mentioned in this study.

Second, transcript‐level evaluation (mRNA expression) was not carried out. Although PCR analysis could provide complementary mechanistic insight, the experimental design prioritized the assessments of protein immunoreactivity (WB), antioxidant enzyme activity (SOD and CAT), and oxidative stress parameters, which reflect directly the functional cellular status. Future studies may include gene expression analyses to further elucidate the underlying regulatory mechanisms.

Third, an appropriate positive control for Co‐resin was not included in the experimental design of this study, which used a complex experimental design that incorporated obesity, supplementation, and sex. Introducing additional groups would have increased the difficulty of data interpretation. In addition, while many studies have evaluated anti‐adiposity effects in MSG‐obese models, few of them have explored potential sex‐specific vulnerabilities in obesity‐related pathologies, as presented herein.

Finally, obesity is a complex pathophysiological state that has been recently submitted to new classification, according to Rubino et al. (2025). Thus, the application of diverse experimental preclinical rodent models to study obesity and to evaluate phytochemical compounds across various metabolic scenarios provided more robust data.

In conclusion, the current study confirmed that hypothalamic‐obese female and male rats present imbalanced plasma and hepatic antioxidant systems that may be involved in hepatic dysfunctions. For the first time, we showed that chronic oral Co‐resin supplementation is able to prevent imbalance in plasma and hepatic antioxidant systems in hypothalamic obese female and male rats, without adiposity reduction.

These protective effects of Co‐resin appear to be more effective in MSG‐obese females, where this response was associated with improvements in triglycerides profile, insulin responsiveness and hepatic histopathology state. Moreover, MSG‐obese rats have many characteristics of MASL found in the initial obese stages, when reversible intervention is possible. Here, we showed that Co‐resin can mitigate plasma and hepatic oxidative imbalances, despite adiposity, favoring oxidative balance. Thus, oral Co‐resin supplementation attenuates or avoids oxidative imbalance common in obesity state and its clinical relevance should be evaluated to strengthen the practical applicability this compounds to treat obesity and associated comorbidities, such as MASL and diabetes.

## Author Contributions

**Sirlei P. Souza:** conceptualization, investigation, writing – original draft, writing – review and editing, methodology, formal analysis, data curation, visualization. **Marina Prigol:** methodology, writing – review and editing, investigation. **Marianela A. F. Urrutia:** methodology, investigation, writing – review and editing. **Beatriz M. Daudt:** methodology, investigation, writing – review and editing. **Sabrina Grassiolli:** conceptualization, writing – review and editing, writing – original draft, supervision, funding acquisition, project administration, resources, data curation, formal analysis, methodology, investigation, visualization. **Caroline M. Aguiar:** methodology, writing – review and editing, investigation. **Gustavo P. Guerra:** methodology, formal analysis, writing – review and editing, data curation, investigation. **Mustafa M. M. Dahleh:** methodology, formal analysis, data curation, writing – review and editing, investigation. **Lucinéia F. C. Ribeiro:** writing – review and editing, methodology, conceptualization, investigation. **Zoé M. N. de Carvalho:** methodology, investigation, data curation, writing – review and editing. **Ellen C. Z. Gomes:** writing – review and editing.

## Funding

This study was financed in part by the Coordenação de Aperfeiçoamento de Pessoal de Nível Superior (CAPES), Brazil, Finance Code 88887.695630/2022‐0. This manuscript is part of the doctoral thesis developed in the Graduate Program in Biosciences and Health and at the Laboratory of Endocrine Physiology and Metabolism.

## Conflicts of Interest

The authors declare no conflicts of interest.

## Supporting information


**Table S1:** Effects of copaiba oil supplementation on liver (Kupffer cell and Nucleic number) and degree of steatosis in MSG‐obese and non‐obese female supplemented with Co.
**Table S2:** Effects of copaiba oil supplementation on liver (Kupffer cell and Nucleic number) and degree of steatosis in MSG‐obese and non‐obese male supplemented with Co.
**Figure S1:** Uncropped Western blots of SOD and CAT enzymes in plasma and liver of males and females.
**Figure S2:** Uncropped Western blots of loading control anti‐mouse β‐actina, for liver and plasma samples from males and females.

## Data Availability

The data that support the findings of this study are available from the corresponding author upon reasonable request.
