# Supplementary material for: Oral Copaiba Oil‐Resin Supplementation Reduces Plasma and Hepatic Oxidative Stress in Female and Male Hypothalamic‐Obese Animals
Source: Food Sci Nutr. 2026 Sep 8;14(9):e72195. doi: 10.1002/fsn3.72195 (PMC13551488; doi:10.1002/fsn3.72195)
Supplement: Supplementary file 1 [file FSN3-14-e72195-s001.docx]

**SUPPLEMENTARY DATA**

**Supplementary Table S1 - Effects of copaiba oil supplementation on liver (Kupffer cell and Nucleic number) and degree of steatosis in MSG-obese and non-obese female supplemented with Co**

|  | **Groups** | | | | | | | | | |  | | **(p-value)** | |  |
| --- | --- | --- | --- | --- | --- | --- | --- | --- | --- | --- | --- | --- | --- | --- | --- |
|  | | **CON_NS_** |  | **MSG_NS_** | |  | **CON_Co_** | |  | **MSG_Co_** | | **I** | | **Co** | **MSG** |
| Kupffer cell | | 82.4±13.72 |  | 90.5±9.88 | |  | 86.2±8.07 | |  | 84.12±11.69 | | 0.2550 | | 0.7703 | 0.4963 |
| Nucleic  number | | 158.4±11.78 |  | 168.75±28.14 | |  | 162.9±25.15 | |  | 150.12±13.70 | | 0.1889 | | 0.4167 | 0.8883 |
| Degree of Steatosis | | 0 |  | 1 |  | | 1 |  | | 1 | | - | | - | - |

**Kupffer cell, nucleic number and degree of hepatic steatosis.** (n 4-10 rats per group). Data presented as percentage (%). Where < 5 % (grade 0); 5 to 33% (grade 1); > 33 to 66% (grade 2) and > 66% (grade 3).

**Supplementary Table S2 - Effects of copaiba oil supplementation on liver (Kupffer cell and Nucleic number) and degree of steatosis in MSG-obese and non-obese male supplemented with Co**

|  | **Groups** | | | | | | | | | | | | | |  | **(p-value)** |  |
| --- | --- | --- | --- | --- | --- | --- | --- | --- | --- | --- | --- | --- | --- | --- | --- | --- | --- |
|  | | **CON_NS_** |  | | **MSG_NS_** | |  | **CON_Co_** | | |  | | **MSG_Co_** | | **I** | **Co** | **MSG** |
| Kupffer cell | | 85.4±8.41 | |  | | 90.75±20.76 | | |  | 84.5±10.28 | |  | | 86±7.87 | 0.6917 | 0.5614 | 0.4822 |
| Nucleic number | | 141.4±14.28 | |  | | 160.75±27.73 | | |  | 141±24.99 | |  | | 131±16.50 | 0.1122 | 0.1033 | 0.6029 |
| Degree of Steatosis | | 0 | |  | | 1 | | |  | 0 | |  | | 0 | - | - | - |

**Kupffer cell, nucleic number and degree of hepatic steatosis.** (n 4-10 rats per group). Data presented as percentage (%). Where < 5 % (grade 0); 5 to 33% (grade 1); > 33 to 66% (grade 2) and > 66% (grade 3).

**FIGURE S1**

**Supplementary Figure S1**: Uncropped Western blots of SOD and CAT enzymes in plasma and liver of males and females

**FIGURE S2**

**Supplementary Figure S2**: Uncropped Western blots of loading control anti-mouse β-actina, for liver and plasma samples from males and females
